# Distinctive Oculomotor Behaviors in Alzheimer's Disease and Frontotemporal Dementia

**DOI:** 10.3389/fnagi.2020.603790

**Published:** 2021-02-04

**Authors:** Carmen Lage, Sara López-García, Alexandre Bejanin, Martha Kazimierczak, Ignacio Aracil-Bolaños, Alberto Calvo-Córdoba, Ana Pozueta, María García-Martínez, Andrea Fernández-Rodríguez, María Bravo-González, Julio Jiménez-Bonilla, Ignacio Banzo, Juan Irure-Ventura, Jordi Pegueroles, Ignacio Illán-Gala, Juan Fortea, Eloy Rodríguez-Rodríguez, Alberto Lleó-Bisa, Cecilia E. García-Cena, Pascual Sánchez-Juan

**Affiliations:** ^1^Institute for Research Marqués de Valdecilla (IDIVAL), University of Cantabria and Department of Neurology, Marqués de Valdecilla University Hospital, Santander, Spain; ^2^Centro de Investigación Biomédica en Red sobre Enfermedades Neurodegenerativas (CIBERNED), Madrid, Spain; ^3^Sant Pau Memory Unit, Department of Neurology, Hospital de la Santa Creu i Sant Pau - Biomedical Research Institute Sant Pau (IIB Sant Pau), Universitat Autonoma de Barcelona, Barcelona, Spain; ^4^Escuela Técnica Superior de Ingeniería y Diseño Industrial – Centre for Automation and Robotics, Technical University of Madrid (UPM) – Consejo Superior de Investigaciones Científicas and Aura Innovative Robotics SL, Madrid, Spain; ^5^Department of Nuclear Medicine, Marqués de Valdecilla University Hospital, Santander, Spain; ^6^Department of Immunology, Marqués de Valdecilla University Hospital, Santander, Spain

**Keywords:** oculomotor, Alzheimer's disease, frontotemporal dementia, biomarkers, antisaccade, smooth pursuit, semantic dementia

## Abstract

Oculomotor behavior can provide insight into the integrity of widespread cortical networks, which may contribute to the differential diagnosis between Alzheimer's disease and frontotemporal dementia. Three groups of patients with Alzheimer's disease, behavioral variant of frontotemporal dementia (bvFTD) and semantic variant of primary progressive aphasia (svPPA) and a sample of cognitively unimpaired elders underwent an eye-tracking evaluation. All participants in the discovery sample, including controls, had a biomarker-supported diagnosis. Oculomotor correlates of neuropsychology and brain metabolism evaluated with 18F-FDG PET were explored. Machine-learning classification algorithms were trained for the differentiation between Alzheimer's disease, bvFTD and controls. A total of 93 subjects (33 Alzheimer's disease, 24 bvFTD, seven svPPA, and 29 controls) were included in the study. Alzheimer's disease was the most impaired group in all tests and displayed specific abnormalities in some visually-guided saccade parameters, as pursuit error and horizontal prosaccade latency, which are theoretically closely linked to posterior brain regions. BvFTD patients showed deficits especially in the most cognitively demanding tasks, the antisaccade and memory saccade tests, which require a fine control from frontal lobe regions. SvPPA patients performed similarly to controls in most parameters except for a lower number of correct memory saccades. Pursuit error was significantly correlated with cognitive measures of constructional praxis and executive function and metabolism in right posterior middle temporal gyrus. The classification algorithms yielded an area under the curve of 97.5% for the differentiation of Alzheimer's disease vs. controls, 96.7% for bvFTD vs. controls, and 92.5% for Alzheimer's disease vs. bvFTD. In conclusion, patients with Alzheimer's disease, bvFTD and svPPA exhibit differentiating oculomotor patterns which reflect the characteristic neuroanatomical distribution of pathology of each disease, and therefore its assessment can be useful in their diagnostic work-up. Machine learning approaches can facilitate the applicability of eye-tracking in clinical practice.

## Introduction

One might think that through the assessment of eye movements we are just evaluating purely motor responses. Far from this, oculomotor behavior is controlled by a widespread and highly interconnected cortical network that works in a hierarchical manner integrating perception, action planning and response generation processes [for a review, see (McDowell et al., 2008; Coiner et al., 2019)]. The simplest ocular movements are visually-guided responses to keep an object of interest fixed in the fovea, either a rapid gaze shift to an appearing stimulus or prosaccade, either a pursuit movement which allows tracking a moving target. Such visually-guided ocular movements are controlled by a core frontoparietal network that includes the frontal and supplementary eye fields and diverse parietal regions like the intraparietal sulcus and superior parietal cortex, and which is supported by subcortical structures and finally connected to the superior colliculus, the saccade generator. In contrast, volitional saccades are ocular responses according to contextual commands, as occurs in the antisaccade or memory saccade paradigms, which require the recruitment of additional cortical regions to carry out and control the execution of an internally-generated plan. In the antisaccade paradigm, the subject is instructed to look at the mirror position of the appearing stimulus. This test has been considered a sensitive measure of inhibitory control, since it requires to withhold the automatic response of looking toward the stimulus and, instead, to generate a saccade in the opposite direction (Heuer et al., 2013). Antisaccade generation is controlled by the same basic frontoparietal network with an additional role of the dorsolateral prefrontal cortex (DLPFC) and anterior cingulate cortex (Munoz and Everling, 2004; Pa et al., 2014). Similarly, the memory saccade paradigm is another cognitively demanding task in which spatial working memory is necessary to retain the precise location where the stimulus has previously appeared and to redirect the gaze to it based on an internal representation. In this case, neuroimaging and lesion studies point to a recruitment activation of DLPFC together with basal ganglia and thalamocortical circuitries (Brown et al., 2004).

When assessing visually-guided and volitional saccades in clinical practice, oculomotor responses can be characterized through diverse features. Although some of them can be described qualitatively through clinical examination, modern video-oculography technology allows to reliably quantify them and to obtain additional parameters based on raw data. Thus, this new technology emerges as a potential tool to measure the integrity of the brain regions that support all these oculomotor features.

Different oculomotor changes have been described in degenerative dementias. In Alzheimer's disease, the most characteristic findings are an increment in saccade latencies and in the antisaccade error rate (Molitor et al., 2015; Kahana Levy et al., 2018). Studies including frontotemporal dementia (FTD) patients are scarcer and some of their results conflicting, which could be due to methodological differences but also to sample heterogeneity between studies, since, apart from one work which described patients with autopsy-confirmed diagnosis (Boxer et al., 2012), no others have exclusively included patients with either pathology or biomarker-supported diagnoses.

We hypothesized that oculomotor parameters that are related to visuospatial functioning and therefore predominantly controlled by posterior brain regions would be significantly impaired in Alzheimer's disease patients, where the hallmark is parietal and posterior temporal atrophy. On the other hand, we would expect that, in patients with the behavioral variant of FTD (bvFTD), the most impaired parameters would be those related to executive functions and which involve supporting activity from frontal lobe regions; while overall performance would be preserved in the semantic variant of primary progressive aphasia (svPPA), where neurodegeneration tends to affect selectively anterior temporal lobes and cognitive functions others than semantic memory are initially less affected.

Our first aim was to describe the oculomotor behavior in response to a wide range of tests in a well-phenotyped sample of patients with Alzheimer's disease, bvFTD and svPPA diagnoses, supported by neuropsychological, neuroimaging and Alzheimer's disease core biomarkers, and to compare it with a sample of cognitively unimpaired controls with negative Alzheimer's disease biomarkers. Our second aim was to analyze whether oculomotor parameters were correlated with the results of neuropsychological assessments and brain metabolism evaluated with 18F-FDG PET to explore if they reflected specific patterns of brain dysfunction. Finally, we tested whether a machine learning classification algorithm could contribute to their differential diagnosis.

## Materials and Methods

### Participants

Three groups of patients with Alzheimer's disease, bvFTD and svPPA were recruited from the Cognitive Disorders Unit of the Marqués de Valdecilla University Hospital (Santander, Spain). Participants were evaluated with the Global Deterioration Scale (Reisberg et al., 1982) and Mini-Mental State Examination (MMSE) (Folstein et al., 1975) as global measures of disease severity. Only patients in a mild dementia stage (Global Deterioration Scale = 4) were included. All Alzheimer's disease patients displayed the classical amnestic phenotype. Diagnoses were established according to consensus criteria for probable Alzheimer's disease (McKhann et al., 2011), bvFTD (Rascovsky et al., 2011), and svPPA (Gorno-Tempini et al., 2011). In addition to congruent neuropsychological and neuroimaging findings (brain CT and/or MRI), all diagnoses were supported by at least one type of biomarker, amyloid-PET, and/or CSF Alzheimer's disease core biomarkers. Final diagnoses were agreed in multidisciplinary meetings including four neurologists (PSJ, ERR, SLG, and CL) and two neuropsychologists (MGM and AP). To minimize misclassification or heterogeneity due to co-pathology, only those cases with a complete concordance between clinicians' diagnosis and biomarker results were included.

Healthy volunteers were participants from the Valdecilla Study of Memory and Brain Aging, a prospective cohort recruiting community-dwelling non-demented people older than 55 years. The baseline protocol includes a comprehensive neuropsychological assessment, brain MRI and CSF analysis of Alzheimer's disease core biomarkers. Those subjects selected for our study had no cognitive complaints and showed normal results in all baseline evaluations, including normal levels of CSF biomarkers, which allows excluding the influence of preclinical Alzheimer's disease on their oculomotor performance.

These four groups were used as a discovery sample for the description of oculomotor performance, exploring neuropsychological and brain metabolic correlates and training machine learning algorithms. The classification accuracy of these algorithms was subsequently tested in two independent samples, a sample of 15 patients with Alzheimer's disease from our center who did not have a biomarker-supported diagnosis; and a sample of 6 bvFTD patients from an independent center, the Memory Unit of Santa Creu i Sant Pau Hospital (Barcelona, Spain) (Alcolea et al., 2019a; Illán-Gala et al., 2019). Both samples were evaluated with the same eye-tracker and an identical examination protocol as the discovery sample.

The study was approved by both local Ethics Committees and all participants gave their written informed consent according to the Declaration of Helsinki. For those patients who could not give a reliable informed consent due to their degree of cognitive impairment, it was obtained from their accompanying relative.

### Oculomotor Evaluation: Procedure and Paradigms

Eye movement recordings were carried out with OSCANN, an eye-tracking device based on video-oculography technology (Hernández et al., 2018). Stimuli were bright green dots with a diameter of two centimeters presented on a display at a viewing distance of 60 centimeters. An examination protocol establishing the sequence of tests and standardized instructions for participants was followed to minimize variability as much as possible. Definite trials were preceded by practice trials that allowed confirming the understanding of the tests. Each definite test was preceded by a nine point-calibration and began with a central fixation target. The evaluation comprised a prosaccade test, an antisaccade test, a memory saccade test and a sinusoidal smooth pursuit test. The prosaccade, antisaccade and memory saccade tests included 12 trials in the horizontal plane followed by eight trials in the vertical plane each. Horizontal trials consisted of the random appearance of targets at 5, 10, or 20 degrees to right or left; and, in vertical trials, at 5 or 12 degrees up or down. The sinusoidal smooth pursuit test included six horizontal trials and six vertical trials.

#### Prosaccade Test

Prosaccades were evaluated by the random appearance of an eccentric target, subsequently replaced by the reappearance of the central target. Subjects were instructed to keep their gaze fixed on the target.

#### Sinusoidal Smooth Pursuit Test

Here, the target moved from one end of the screen to the other and subjects were asked to follow it as accurately as possible.

#### Antisaccade Test

In a similar fashion to that in the prosaccade test, the central target was replaced by the appearance of an eccentric target, but the command, in this case, was: “When the target appears at one side, look at the opposite location, in a mirrored way. If you realize that you have looked at the target, try to correct yourself looking at the opposite location.”

#### Memory Saccade Test

As in the prosaccade test, the target appeared eccentrically and then at the central position. After that, the target disappeared, leaving the screen blank. The instructions were: “Keep your gaze fixed on the target when it appears at one side and when it comes back to the center. When the central target disappears, look at the location where it had previously appeared.”

#### Oculomotor Parameters

In order to use the subtle alterations of eye movements for diagnostic aims, it is crucial to guarantee the reproducibility of the measuring, which is described in the OSCANN medical device user manual and summarized in a related publication (Hernández et al., 2018). After the automatic analysis of the images captured by the eye-tracker camera, we extracted features from each oculomotor test following the published methodology (García Cena et al., 2020).

Oculomotor responses can be characterized by diverse parameters. For descriptive purposes, such parameters have been grouped into three domains: (a) parameters related to spatial accuracy, as saccade error (the deviation of the final position of the gaze from the target, measured as positive or negative error) and pursuit error (the difference between the target position and the gaze position during a pursuit test); (b) parameters related to time, as latency (defined by the time delay between the appearance of a peripheral target and the onset of the ocular movement) and pursuit gain (the rate between ocular velocity and target velocity during a pursuit test); and (c) parameters related to success, as the percentage of correct memory saccades in the memory saccade test, and, in the antisaccade test, the percentage of correct antisaccades, corrected erroneous antisaccades (henceforth, corrected antisaccades) and successful antisaccades, which represent the sum of correct and corrected antisaccades. Precise definitions can be found in the Supplementary Material.

### Neuropsychological Evaluation

We hypothesized that oculomotor parameters would be correlated with those cognitive domains that are directly implicated in the performance of each oculomotor response or, indirectly, which are theoretically controlled by the same brain region that supports that oculomotor feature, including visuospatial function, memory and executive function. Therefore, we designed a neuropsychological evaluation that assessed verbal memory with the Free and Cued Selective Reminding Test (Pena-Casanova et al., 2009a), visual memory with the Rey-Osterrieth Complex Figure Test (ROCFT) Free Delayed Recall (Pena-Casanova et al., 2009a), constructional praxis with ROCFT Copy (Pena-Casanova et al., 2009a), ideomotor apraxia with imitation of finger gestures (Pena-Casanova, 2005), visuospatial ability with the Number Location subtest of the Visual Object and Space Perception Battery (Pena-Casanova et al., 2009b) and attention and executive function with the Trail Making Test (TMT) A and B and Symbol digit test (Pena-Casanova et al., 2009c).

### Biomarker Studies

For the CSF study, levels of amyloid-β (Aβ1-42), total tau and phosphorylated tau (p-tau181) were quantified with the LUMIPULSE G600II automated platform (Fujirebio) and interpreted according to established cut-off points (Alcolea et al., 2019b).

A subset of patients underwent a neuroimaging study including a 2-[18F] Fluoro-2-Deoxy-D-Glucose (18F-FDG) PET and/or an amyloid-PET with Pittsburgh Compound-B (PiB)/CT scan, obtained within 1-week interval using a Siemens Biograph LSO Pico 3D equipment (Siemens Healthcare Molecular Imaging, Hoffman Estates, Illinois, USA). Participants were injected with 3-4 MBq/kg 18F-FDG and 555 MBq of 11C-PiB. Image acquisition consisted of one static image acquired from 30 to 45 min after injection for 18F-FDG PET and from 60 to 90 min for 11C-PiB PET. The information provided by the CT was used to correct the attenuation and images were reconstructed on a 128 × 128 matrix using the ordered subsets expectation maximization iterative method.

11C-PiB PET was evaluated exclusively by visual read as positive or negative cortical amyloid-β deposition. To investigate the metabolic correlates of oculomotor responses, we preprocessed the 18F-FDG data using Statistical Parametric Mapping version 12 (SPM12) software (Well-come Department of Imaging Neuroscience, Institute of Neurology, London, UK) implemented in MATLAB 9.2 (The MathWorks, Sherborn, MA). Images were quantitatively normalized using the pons-vermis as the reference region, spatially normalized to the Montreal Neurological Institute PET template, and smoothed with a Gaussian kernel of full width at half maximum 8 mm. All resulting images were visually inspected to check for possible registration errors.

### Statistical Analysis

Oculomotor parameters were compared across the four diagnostic groups of the discovery sample by ANOVA with Tukey's *post hoc* test. Multivariate analyses using General Linear Models with the oculomotor parameter as the dependent variable and age and sex as covariates were performed. The influence of neurodepressant drugs on oculomotor performance was investigated as a potential confounding variable. Spearman's test was used to look for significant correlations between each oculomotor parameter and the number of sedatives taken by a patient, which included antidepressants, benzodiazepines, neuroleptics or antiepileptics. In those cases where the level of significance was <0.1, the number of drugs was included in the multivariate analysis. Differences between groups were considered significant when *P* < 0.05. Analyses were performed using The Statistical Packages for Social Sciences (SPSS 19.0.1).

The most significant oculomotor parameter from each test was selected to explore its cognitive and brain metabolic correlates. Neuropsychological scores were compared with these parameters with a Spearman's correlation and linear regression analyses with age as a covariate. Multiple regressions were performed voxel-wise to assess the relationship between 18F-FDG standard uptake value rate and the selected oculomotor parameters across the whole gray matter. In these models, we regressed out the time delay between the PET acquisition and the oculomotor evaluation. A threshold of *P* < 0.001 uncorrected, together with a cluster extent k>100 mm3, was used for all these analyses.

### Machine Learning Classification Algorithms

Our third aim was to construct three classification algorithms using a machine learning approach for the differentiation between (1) Alzheimer's disease patients and controls; (2) bvFTD patients and controls; (3) Alzheimer's disease and bvFTD patients. Due to its small size, the svPPA sample was not considered suitable for this analysis. Only the data from the discovery sample was used for generating the classification algorithms.

The data set used in any machine learning classifier must be carefully prepared. Firstly, a normalization was performed to avoid dispersion in data with different dimensions. The normalization allows the different dimensions of the data to be scaled to standardize the range of the characteristics, since it can affect the results in a critical way (Graf and Borer, 2001).

$$\begin{array}{l} \hat{x}=\frac{x-\bar{x}}{\sigma } \end{array}$$

Where *x* is the value of a feature, while $\bar{x}$ and σ are the mean value and the standard deviation of the feature set, respectively.

In our study, the number of subjects was less than the number of features, so the partial less square regression (PLSR or PLS) technique was applied to reduce the number of significance variables.

Let's *X* ∈ ℝ^*n*^ the set of independent features and *Y* ∈ ℝ^*n*^ the set of dependent features. The relation between each set is given by a score vector. We compute the score vector using the partial minimum square regression (PLSC) (Krishnan et al., 2011). Then, the feature sets are defined by:

$$\begin{array}{l} X=TP^{T}+E\\ Y=\;UQ^{T}+F \end{array}$$

Where *T* ∈ ℝ^*n*×*p*^, *U* ∈ ℝ^*n*×*p*^ while *P* ∈ ℝ^*N*×*p*^ and *Q* ∈ ℝ^*M*×*p*^ are the weight matrix and *E* ∈ ℝ^*n*×*N*^ *F* ∈ ℝ^*n*×*M*^ are the residual matrices. The PLS2 algorithm was used to compute each matrix.

Finally, the Fisher discriminant ratio (FDR) was used to select the features to train the machine learning algorithm. One of the main advantages of FDR is that we can associate the sets of features with a label such as: Alzheimer's disease, bvFTD or controls. The FDR is defined by the μ_*i*_: and σ_*i*_ are the mean and variance of the set *i*.

$$\begin{array}{l} FDR=\frac{{\left(\mu_{1}-\mu_{2}\right)}^{2}}{\left(\sigma_{1}^{2}-\sigma_{2}^{2}\right)} \end{array}$$

**Figure 1:**
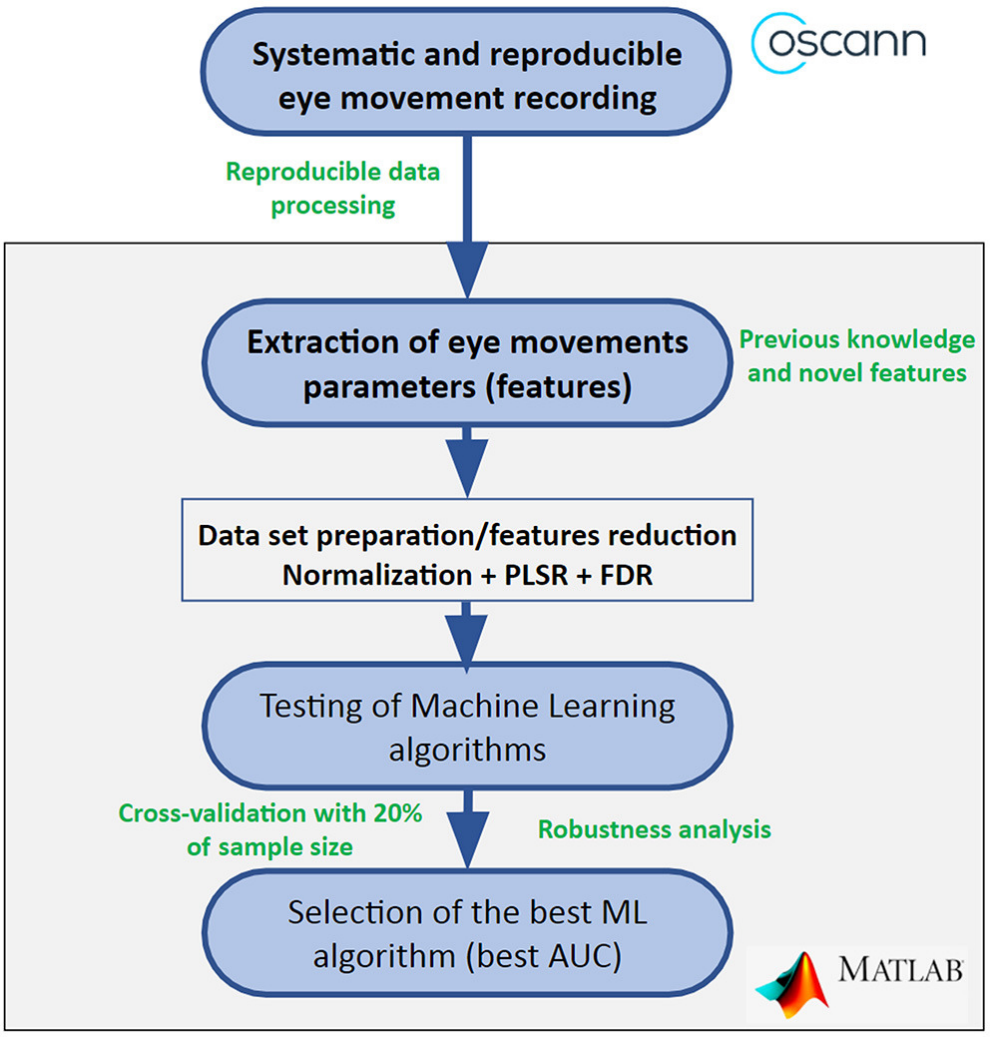
Flow chart to find the most suitable combination between eye movement features and machine learning algorithm. AUC, area under the curve; FDR, Fisher discriminant ratio; ML, machine learning; PLSR, partial less square regression.

Under Matlab environment, we performed multiple tests in order to find the most suitable combination between oculomotor features and type of machine learning algorithm for each pair of groups (Alzheimer's disease patients vs. controls; bvFTD vs. controls; Alzheimer's disease vs. bvFTD), as it is shown in Figure 1. The most suitable combination was defined by the biggest area under the curve (AUC) in the receiver operating characteristic (ROC) curve. The classifiers were modeled through a cross-validation process in which the sample was divided in four subsets (80% of the sample) for training and one subset (20% of the sample) for classification.

**Figure 2:**
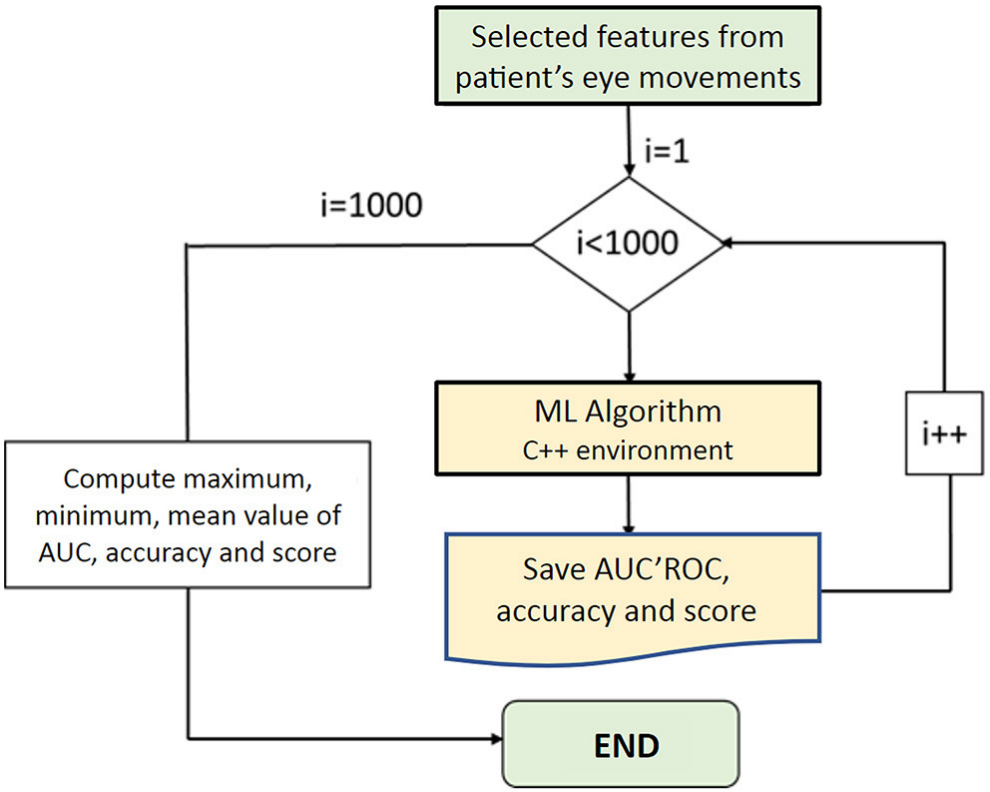
Loop implemented to test the confidence interval of the selected machine learning algorithm. AUC, area under the curve; I, iterations; ML, machine learning; ROC, receiver operating characteristic.

Despite the cross-validation performed by Matlab, we decided to implement the selected algorithm under Microsoft Visual Studio C++ language in order to get a software independent from Matlab and to carry out the cross-validation of the algorithm with the whole set of samples instead of a subset like in the previous case and through a loop of 1,000 iterations. Moreover, under C++ environment, the confidence interval of the algorithm was computed using the following flow chart displayed in Figure 2.

Finally, we were interested in applying the classification algorithms for the differentiation between controls and each dementia group (Alzheimer's disease patients vs. controls and bvFTD vs. controls) in independent samples of patients with the aim of testing their external validity. For doing so, the previously generated algorithms were applied to the independent samples of Alzheimer's disease and bvFTD patients and their classification accuracy was assessed using ROC curves.

## Results

### Demographics

From the initial sample of evaluated subjects, five patients were excluded due to a biomarker result discordant with their clinical group: one subject with a clinical diagnosis of probable Alzheimer's disease dementia due to normal levels of CSF biomarkers and negative PiB-PET; and two bvFTD patients and two svPPA patients due to positivity in PiB-PET. After that, the discovery population included 18 Alzheimer's disease patients, 18 bvFTD, seven svPPA, and 29 controls. Diagnoses were supported by at least one type of Alzheimer's disease core biomarker (11C-PiB PET in 40/43 patients; CSF study in 15/43 patients and 29/29 controls). Groups did not differ in terms of age at evaluation (*P* = 0.36) or disease duration (*P* = 0.65) (Table 1). Significant differences were observed in the MMSE scoring, with the lowest performance in the Alzheimer's disease group, as well as in the intake of potentially sedative drugs, with a higher intake in bvFTD patients compared to the other three groups.

**Table 1:** Demographics and neuropsychological performance.

	**Controls**	**Alzheimer's disease**	**BvFTD**	**SvPPA**	**Group effect *P*-value**	**Alzheimer's disease vs. bvFTD*P*-value**	**Alzheimer's disease vs. svPPA *P*-value**	**BvFTD vs. svPPA*P*-value**
*N*	29	18	18	7				
Age, years	66.21 (5.51)	68.17 (6.96)	68.83 (8.71)	70.86 (8.11)	*0.36*	*0.99*	*0.83*	*0.92*
Gender, % female	79.31	66.67	22.22	57.14	***0.0014***	***0.018***	*0.67*	*0.16*
Disease duration, years	**–**	4.94 (1.73)	5.56 (3.15)	6.00 (3.79)	*0.65*	*0.79*	*0.67*	*0.93*
Intake of sedatives, mean number	0.14 (0.44)	1.06 (0.87)	2.06 (1.11)	0.86 (0.90)	***<0.00001***	***0.0022***	*0.95*	***0.0073***
MMSE (0–30)	28.96 (0.92)	16.72 (5.23)	23.50 (2.73)	22.43 (4.65)	***<0.00001***	***<0.00001***	***0.0015***	*0.89*
FCSRT Total Free and Cued Recall (0–48)	42.96 (5.50)	11.70 (9.11)	30.17 (12.55)	22.00 (10.22)	***<0.00001***	***0.000017***	*0.17*	*0.29*
FCSRT Delayed Free and Cued Recall (0–16)	15.07 (1.36)	2.60 (2.91)	9.67 (4.64)	6.00 (3.74)	***<0.00001***	***<0.00001***	*0.20*	*0.10*
ROCFT Recall (0–36)	16.04 (5.23)	1.63 (3.11)	6.65 (5.18)	7.25 (3.30)	***<0.00001***	*0.091*	*0.25*	*0.10*
ROCFT Copy (0–36)	32.86 (2.85)	20.13 (11.14)	25.06 (9.24)	28.75 (7.09)	***0.000080***	*0.37*	*0.20*	*0.78*
Imitative praxis (0–8)	7.92 (0.27)	6.40 (1.90)	7.73 (0.59)	8.00 (0.00)	***0.00017***	***0.0024***	***0.0076***	*0.93*
VOSP NL (0–10)	9.21 (0.96)	6.50 (2.56)	7.72 (2.49)	8.80 (1.10)	***0.0018***	*0.40*	*0.13*	*0.65*
Trail Making Test A (seconds)	45.93 (12.70)	157.10 (72.12)	119.22 (66.24)	84.80 (25.15)	***<0.00001***	*0.19*	***0.036***	*0.48*
Trail Making Test B (seconds)	103.52 (55.05)	179.50 (95.46)	220.60 (100.36)	145.50 (82.73)	***0.0052***	*0.87*	*0.95*	*0.52*
Symbol digit test	39.55 (14.14)	16.43 (12.33)	19.33 (9.25)	21.60 (10.21)	***<0.00001***	*0.95*	*0.88*	*0.98*

*Mean values of each demographic characteristic and neuropsychological score, with standard deviations in brackets (). Group effect calculated with ANOVA for the four groups. Only post hoc P-values for patient groups are shown. Bold values indicate P < 0.05. BvFTD, behavioral variant frontotemporal dementia; FCSRT, Free and Cued Selective Reminding Test; MMSE, Mini-Mental State Examination; ROCFT, Rey-Osterrieth Complex Figure Test; svPPA, semantic variant of primary progressive aphasia; VOSP NL, Visual Object and Space Perception Battery, Number Location subtest. r, correlation coefficient; P, level of significance*.

### Oculomotor Evaluation

Those oculomotor parameters significantly correlated with the number of sedatives taken by a patient were: (a) in the antisaccade test: correct, corrected and successful antisaccades, horizontal and vertical corrected antisaccade latency, horizontal and vertical positive error, and vertical negative error; (b) in the memory saccade test: correct memory saccades, horizontal and vertical latency and horizontal and vertical negative error. For the analysis of these parameters, the number of drugs was included in the multivariate analysis as a covariate.

#### Parameters Related to Spatial Accuracy

**Figure 3:**
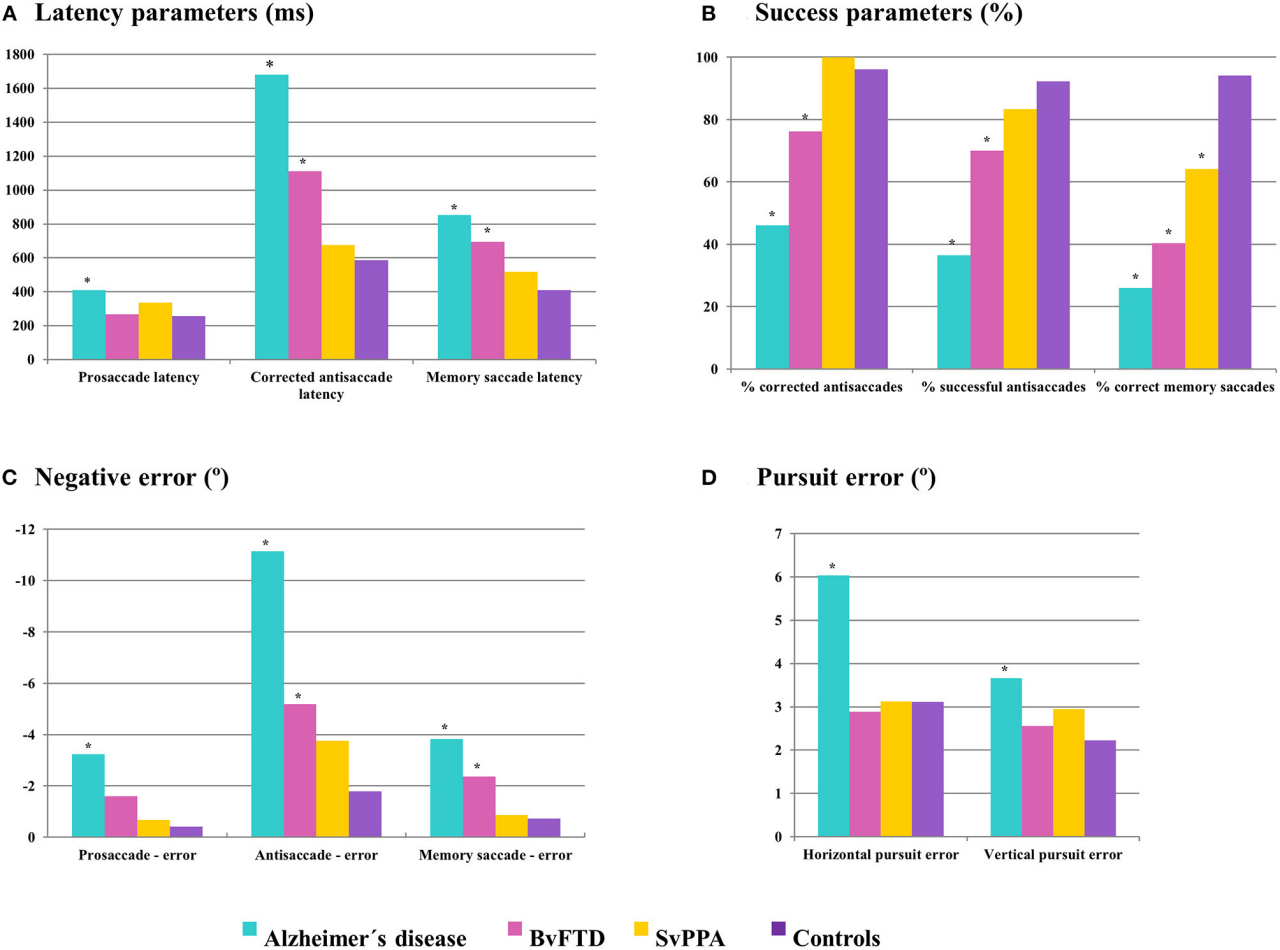
Performance of controls and dementia groups in oculomotor parameters related to time **(A)**, success **(B)**, and spatial accuracy **(C,D)**. Asterisks mark those dementia groups which show significant differences (*P* < 0.05) compared to controls. ms, milliseconds; °, degrees.

The poorest accuracy performance was obtained in the Alzheimer's disease group, who showed a tendency to make hypometric saccades with greater values of negative error in their prosaccades, antisaccades and memory saccades than the other groups, which resulted significantly different from controls in these three tests (Tables 2–**4**; Figure 3). For the most cognitively demanding tasks, the antisaccade and memory saccade tests, the bvFTD group also made less accurate saccades than controls, with significantly greater values of positive and negative error in the antisaccade test and negative error in the memory saccade test.

**Table 2:** Visually-guided responses: Prosaccade test and sinusoidal smooth pursuit test.

**Oculomotor parameter**	**Controls**	**Alzheimer's disease vs. controls**	**BvFTD vs. controls**	**SvPPA vs. controls**	**Alzheimer's disease vs. bvFTD**	**Alzheimer's disease vs. svPPA**	**BvFTD vs. svPPA**
		***Crude P-value (adjusted P-value)***	***Crude P-value (adjusted P-value)***	***Crude P-value (adjusted P-value)***	***Crude P-value (adjusted P-value)***	***Crude P-value (adjusted P-value)***	***Crude P-value (adjusted P-value)***
**Prosaccade latency (ms)**							
Horizontal mean value (SD)	257.50 (52.24)	409.60 (226.60) ***0.0016 (0.00017)***	266.23 (34.20) *0.99 (0.49)*	336.62 (160.48) *0.49 (0.094)*	***0.012 (0.011)***	*0.60 (0.28)*	*0.64 (0.31)*
Vertical mean value (SD)	264.80 (34.70)	315.14 (100.48) *0.099 **(0.014)***	282.79 (42.25) *0.84 (0.18)*	322.63 (119.10) *0.21 **(0.028)***	*0.53 (0.41)*	*1.00 (0.68)*	*0.58 (0.29)*
**Return saccade latency (ms)**							
Horizontal mean value (SD)	250.59 (72.96)	361.06 (202.30) ***0.037 (0.0042)***	245.48 (28.48) *1.00 (0.38)*	353.50 (219.70) *0.27 **(0.046)***	*0.063 (0.12)*	*1.00 (0.95)*	*0.28 (0.24)*
Vertical mean value (SD)	250.19 (36.30)	313.01 (133.12) *0.082 **(0.016)***	280.70 (56.47) *0.64 (0.10)*	327.88 (114.81) *0.14 **(0.025)***	*0.68 (0.62)*	*0.98 (0.62)*	*0.59 (0.38)*
**Positive error (°)**							
Horizontal mean value (SD)	0.67 (0.73)	1.06 (1.43) *0.57 (0.16)*	0.69 (0.62) *1.00 (0.88)*	1.34 (1.08) *0.39 (0.074)*	*0.70 (0.31)*	*0.91 (0.44)*	*0.48 (0.13)*
Vertical mean value (SD)	0.54 (0.39)	0.95 (1.17) *0.25 (0.063)*	0.60 (0.51) *0.99 (0.61)*	0.78 (0.45) *0.85 (0.38)*	*0.48 (0.28)*	*0.95 (0.64)*	*0.94 (0.68)*
**Negative error (°)**							
Horizontal mean value (SD)	−0.41 (0.62)	−3.23 (4.38) ***0.022 (0.0076)***	−1.60 (3.44) *0.61 (0.25)*	−0.68 (0.60) *1.00 (0.95)*	*0.43 (0.26)*	*0.32 (0.088)*	*0.43 (0.44)*
Vertical mean value (SD)	−0.67 (1.44)	−1.55 (2.39) *0.34 (0.15)*	−0.47 (0.37) *0.98 (0.59)*	−0.48 (0.34) *1.00 (0.63)*	*0.20 (0.070)*	*0.53 (0.15)*	*1.00 (0.94)*
**Pursuit gain**							
Horizontal mean value (SD)	0.79 (0.18)	0.89 (0.78) *0.87 (0.53)*	0.87 (0.29) *0.93 (0.95)*	0.67 (0.24) *0.94 (0.45)*	*1.00 (0.63)*	*0.72 (0.26)*	*0.78 (0.44)*
Vertical mean value (SD)	0.75 (0.33)	0.83 (1.02) 0.98 *(1.00)*	0.56 (0.60) *0.78 **(0.031)***	0.73 (0.13) 1.00 *(0.54)*	*0.610 **(0.034)***	*0.99 (0.55)*	*0.95 (0.29)*
**Pursuit error (°)**							
Horizontal mean value (SD)	3.11 (2.01)	6.03 (3.01) ***0.00043 (0.000065)***	2.89 (1.93) *0.99 (0.88)*	3.12 (1.51) *1.00 (0.85)*	***0.00055 (0.00072)***	***0.041 (0.012)***	*1.00 (0.93)*
Vertical mean value (SD)	2.23 (1.29)	3.66 (1.44) ***0.0064 (0.00098)***	2.56 (1.34) 0.87 *(0.18)*	2.95 (1.30) 0.65 *(0.21)*	*0.090 (0.10)*	*0.69 (0.30)*	*0.93 (0.82)*

*Bold values indicate P < 0.05. BvFTD, behavioral variant frontotemporal dementia; ms, milliseconds; SD, Standard deviations; svPPA, semantic variant of primary progressive aphasia; °, degrees. r, correlation coefficient; P, level of significance*.

SvPPA patients did not show significant differences from controls in error values except for a greater positive error in horizontal antisaccades (mean difference 3.25 degrees, *P* = 0.020). Their lower negative error values also distinguished them from Alzheimer's disease and bvFTD both in the antisaccade and the horizontal memory saccade test.

**Figure 4:**
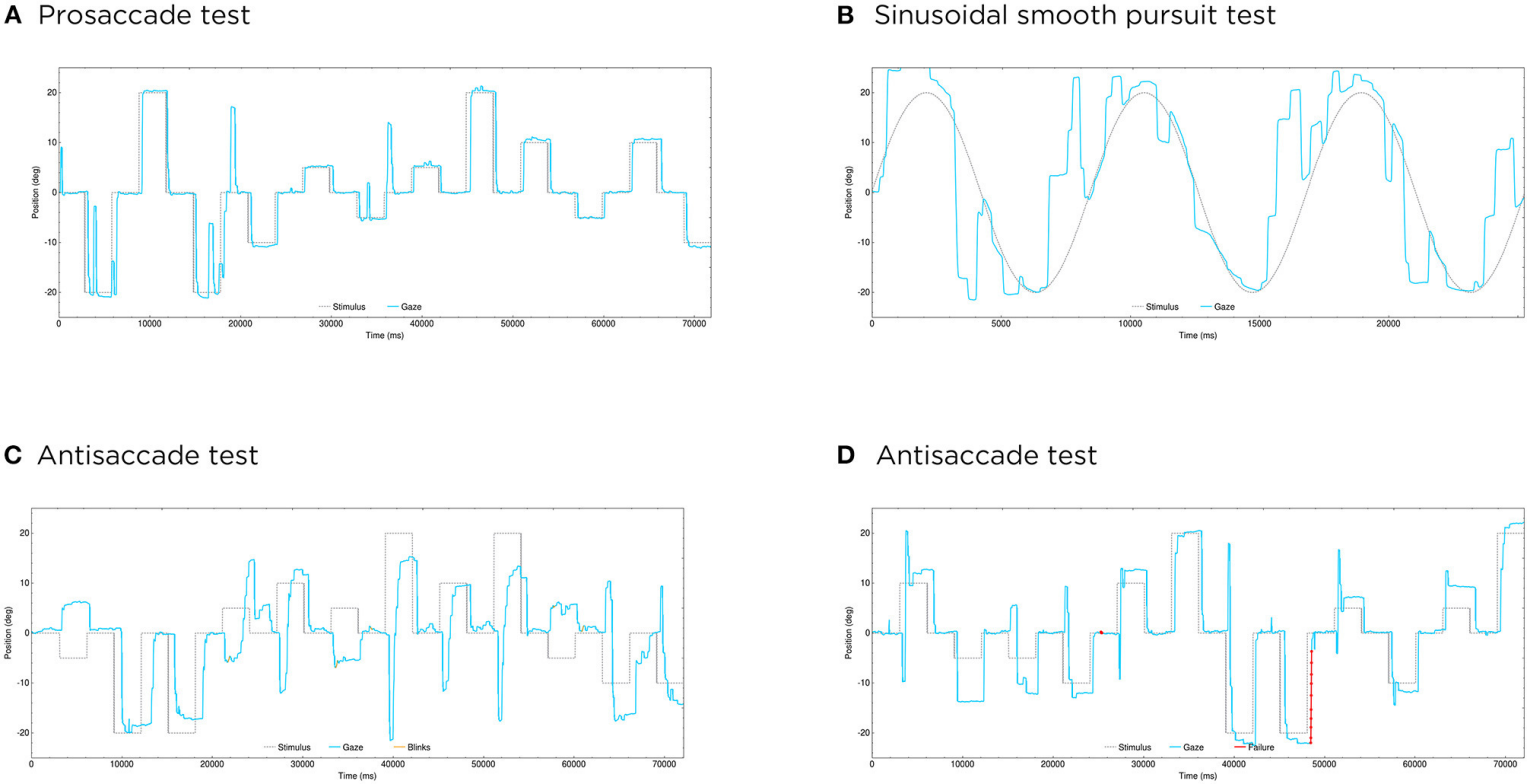
Some clinical examples of oculomotor evaluations. **(A,B)** Show the performance of a patient with Alzheimer's disease in an horizontal prosaccade test **(A)** and an horizontal sinusoidal smooth pursuit test **(B)**, with great difficulties in overlapping his gaze with the moving target in the latter. **(C,D)** Illustrate the different performance in the horizontal antisaccade test between a patient with bvFTD **(C)**, with some successful responses but that are slow and inaccurate; and a patient with svPPA **(D)**, who also makes some errors but followed by fast corrections in this case. In the ordinate axis, 0 indicates the center, positive values the right side and negative values the left side. Blue lines represent the patient's ocular movement and dotted lines the expected location of the gaze, which corresponds to the target position in the prosaccade and sinusoidal smooth pursuit tests, and to the opposite position of the target in the antisaccade test. Blinks are marked in yellow and pupil detection failure (usually also due to blinks) in red. deg, degrees; ms, milliseconds.

In the smooth pursuit test, the greatest pursuit error values were observed in the Alzheimer's disease group, which were significantly different from controls in the horizontal (mean difference 2.92 degrees, *P* = 0.000065) as well as in the vertical test (mean difference 1.43 degrees, *P* = 0.00098) (Figures 3, 4, Table 2). This parameter also distinguished Alzheimer's disease patients from the other two dementia groups in the horizontal test (mean difference 3.14 degrees for bvFTD, *P* = 0.00072; 2.90 degrees for svPPA, *P* = 0.012).

#### Parameters Related to Time

In the prosaccade test, Alzheimer's disease patients showed longer latencies than controls (Table 2), with significant differences in horizontal (mean difference 152.10 ms, *P* = 0.00016) and vertical prosaccades (mean difference 50.34 ms, *P* = 0.014) as well as in return saccades (mean difference for horizontal prosaccades 110.48 ms, *P* = 0.0042; and for vertical prosaccades 62.82 ms, *P* = 0.016). Additionally, svPPA patients displayed significantly longer latencies than controls in vertical prosaccades and return saccades. Conversely, prosaccade latencies in bvFTD patients were comparable to controls, which distinguished them from the Alzheimer's disease group in the horizontal test (mean difference 143.37 ms, *P* = 0.011).

Alzheimer's disease patients also showed the longest latencies for corrected antisaccades and memory saccades, in this case followed by bvFTD, which could differentiate both groups from controls, in the horizontal as well as in the vertical plane (Tables 3, 4). In these tests, svPPA latencies were similar to controls and the longer latencies in Alzheimer's disease patients made it possible to differentiate the two dementia groups in both corrected antisaccades and memory saccades.

**Table 3:** Volitional saccades: Antisaccade test.

**Oculomotor parameter**	**Controls**	**Alzheimer's disease vs. controls**	**BvFTD vs. controls**	**SvPPA vs. controls**	**Alzheimer's disease vs. bvFTD**	**Alzheimer's disease vs. svPPA**	**BvFTD vs. svPPA**
		***Crude P-value (adjusted P-value)***	***Crude P-value (adjusted P-value)***	***Crude P-value (adjusted P-value)***	***Crude P-value (adjusted P-value)***	***Crude P-value (adjusted P-value)***	***Crude P-value (adjusted P-value)***
**% Correct antisaccades (SD)**	26.38 (18.75)	4.00 (5.41) ***0.00031 (0.00037)***	7.50 (10.80) ***0.0022 (0.018)***	25.00 (29.33) *1.00 (0.85)*	*0.93 (0.85)*	***0.045 (0.016)***	*0.12 **(0.038)***
**% Corrected antisaccades (SD)**	96.06 (11.11)	46.08 (34.94) ***1.61 × 10**^**−6**^**(1.64 × 10**^**−6**^**)***	76.15 (33.96) *0.057 **(0.026)***	100.00 (0.00) *0.98 (0.88)*	***0.0066 (0.0496)***	***0.00016 (0.000058)***	*0.19 **(0.026**)*
**% Successful antisaccades (SD)**	92.24 (14.92)	36.44 (30.77) ***1.36 × 10**^**−7**^**(8.75 × 10**^**−8**^**)***	70.00 (34.88) ***0.030 (0.0072)***	83.33 (16.63) *0.86 (0.26)*	***0.0024 (0.047)***	***0.0015 (0.00065)***	*0.69 (0.12)*
**Corrected antisaccade latency (ms)**							
Horizontal mean value (SD)	587.14 (183.32)	1680.38 (879.79) ***0.000012 (2.50 × 10**^**−06**^**)***	1112.34 (959.28) *0.056 **(0.0024)***	676.84 (173.65) *0.99 (0.24)*	*0.093 (0.31)*	***0.0094 (0.0031)***	*0.48 (0.056)*
Vertical mean value (SD)	550.64 (90.90)	1485.63 (1066.28) ***0.000055 (0.0000020)***	937.94 (564.51) *0.18 **(0.0053)***	560.45 (139.67) *1.00 (0.38)*	*0.071 (0.33)*	***0.0090 (0.0034)***	*0.53 (0.054)*
**Antisaccade positive error (°)**							
Horizontal mean value (SD)	2.43 (2.73)	4.56 (3.70) *0.36 (0.069)*	8.40 (3.41) ***5.20 × 10**^**−05**^**(0.00019)***	5.69 (4.25) *0.12 **(0.020)***	***0.048 (0.013)***	*0.92 (0.51)*	*0.33 (0.081)*
Vertical mean value (SD)	1.72 (1.40)	4.87 (2.72) ***0.000033 (0.000018)***	3.46 (1.26) ***0.022 (0.041)***	2.50 (1.62) *0.74 (0.27)*	*0.22 (0.17)*	***0.043 (0.013)***	*0.67 (0.28)*
**Antisaccade negative error (°)**							
Horizontal mean value (SD)	−1.79 (1.10)	−11.15 (10.66) ***0.00099 (0.00013)***	−5.18 (9.81) *0.42 **(0.046)***	−3.76 (1.94) *0.92 (0.47)*	*0.12 (0.14)*	*0.14 **(0.035)***	*0.97 (0.38)*
Vertical mean value (SD)	−1.58 (0.66)	−5.88 (5.32) ***0.019 (0.0042)***	−5.01 (5.99) ***0.047 (0.039)***	−1.48 (0.73) *1.00 (1.00)*	*0.95 (0.97)*	*0.11 **(0.026)***	*0.22 **(0.037)***

*Bold values indicate P < 0.05. BvFTD, behavioral variant frontotemporal dementia; ms, milliseconds; SD, Standard deviations; svPPA, semantic variant of primary progressive aphasia; °, degrees. r, correlation coefficient; P, level of significance*.

**Table 4:** Volitional saccades: Memory saccade test.

**Oculomotor parameter**	**Controls**	**Alzheimer's disease vs. controls**	**BvFTD vs. controls**	**SvPPA vs. controls**	**Alzheimer's disease vs. bvFTD**	**Alzheimer's disease vs. svPPA**	**BvFTD vs. svPPA**
		***Crude P-value (adjusted P-value)***	***Crude P-value (adjusted P-value)***	***Crude P-value (adjusted P-value)***	***Crude P-value (adjusted P-value)***	***Crude P-value (adjusted P-value)***	***Crude P-value (adjusted P-value)***
**% Correct memory saccades (SD)**	94.14 (9.74)	26.00 (19.41) ***2.71 × 10**^**−11**^**(1.22 × 10**^**−09**^**)***	40.36 (25.15) ***1.01 × 10**^**−09**^**(0.00064)***	64.17 (36.94) ***0.0065 (0.025)***	*0.30 **(0.014)***	***0.0022 (0.00019)***	*0.072 (0.14)*
**Memory saccade latency (ms)**							
Horizontal mean value (SD)	410.26 (197.59)	852.58 (314.01) ***0.00059 (0.000069)***	695.44 (343.24) ***0.014 (0.0029)***	517.37 (245.48) *0.83 (0.18)*	*0.55 (0.92)*	*0.12 **(0.041)***	*0.58 (0.068)*
Vertical mean value (SD)	384.39 (115.94)	1163.14 (903.67) ***0.00043 (0.00088)***	866.05 (461.76) ***0.0076** (0.26)*	492.58 (332.35) *0.96 (0.92)*	*0.46 (0.14)*	*0.056 **(0.0093)***	*0.38 (0.26)*
**Memory saccade positive error (°)**							
Horizontal mean value (SD)	1.48 (0.76)	2.53 (3.39) *0.39 (0.11)*	1.38 (1.37) *1.00 (0.88)*	1.53 (0.75) *1.00 (0.86)*	*0.49 (0.25)*	*0.67 (0.29)*	*1.00 (0.97)*
Vertical mean value (SD)	0.98 (0.56)	1.44 (1.71) *0.75 (0.32)*	0.91 (0.64) *1.00 (0.89)*	0.88 (0.44) *1.00 (0.84)*	*0.73 (0.32)*	*0.77 (0.34)*	*1.00 (0.94)*
**Memory saccade negative error (°)**							
Horizontal mean value (SD)	−0.72 (0.67)	−3.82 (2.11) ***0.000019 (2.14 × 10**^**−06**^**)***	−2.37 (1.91) ***0.011 (0.00019)***	−0.86 (0.54) *1.00 (0.20)*	*0.13 (0.42)*	***0.0025 (0.00032)***	*0.18 **(0.0055)***
Vertical mean value (SD)	−0.98 (1.37)	−2.17 (1.30) *0.34 (0.27)*	−2.30 (1.47) *0.14 (0.095)*	−1.41 (1.52) *0.94 (0.73)*	*1.00 (0.39)*	*0.85 (0.57)*	*0.73 (0.15)*

*Bold values indicate P < 0.05. BvFTD, behavioral variant frontotemporal dementia; ms, milliseconds; SD, Standard deviations; svPPA, semantic variant of primary progressive aphasia; °, degrees. r, correlation coefficient; P, level of significance*.

In the smooth pursuit test (Table 2), bvFTD patients presented the lowest vertical gain values, showing significant differences with controls (mean difference 0.19, *P* = 0.031) and Alzheimer's disease patients (mean difference 0.27, *P* = 0.034), while there were no clear differences in the horizontal pursuit.

#### Parameters Related to Success

For these parameters, the performance between horizontal and vertical trials was compared. There were no statistically significant differences in the percentage of correct, corrected and successful antisaccades nor correct memory saccades, so horizontal and vertical saccades were pooled to obtain total scores for each parameter to increase statistical power and simplify results. In previous parameters related to time and accuracy, we considered that keeping horizontal and vertical results disaggregated was more appropriate since the amplitudes to the target in horizontal and vertical trials were not the same and this might impact values related to time or distance.

Again, the Alzheimer's disease group showed the poorest performance, with significantly lower percentages of corrected and successful antisaccades and correct memory saccades than the other three groups (Tables 3, 4). Although not as low as Alzheimer's disease patients, the bvFTD group also obtained significantly lower percentages of correct, corrected, and successful antisaccades as well as correct memory saccades than controls. There were no significant differences between Alzheimer's disease and bvFTD in the percentage of correct antisaccades, but bvFTD patients were able to make a higher number of corrections than Alzheimer's disease patients, and this made that bvFTD patients obtained a significantly higher percentage of successful antisaccades (mean difference 33.56%, *P* = 0.047). Additionally, the bvFTD group was superior to Alzheimer's disease in the percentage of correct memory saccades (mean difference 14.36%, *P* = 0.014).

In the antisaccade test, the performance of svPPA patients was comparable to controls and this distinguished them from the Alzheimer's disease and bvFTD groups in the percentage of correct and corrected antisaccades (Figure 4). However, at the memory saccade test, svPPA patients performed better than the other two dementia groups but still significantly worse than controls (mean difference 29.97%; *P* = 0.025).

### Relationship Between Oculomotor Behavior and Neuropsychological Performance

As expected, significant differences across the four groups were found for all neuropsychological tests, with the highest performance in controls (Table 1). Alzheimer's disease patients performed significantly worse than bvFTD in verbal memory and imitative praxis. No significant differences were found between bvFTD and svPPA patients.

For the comparison with cognitive and brain metabolic results, we selected the oculomotor parameter with the lowest *P*-value from each test, which were: horizontal prosaccade latency, horizontal pursuit error and percentage of correct memory saccades. In the antisaccade test, the percentage of successful antisaccades showed the lowest *P-*value, but, since this is a composite measure, it was not considered an optimal parameter to investigate for correlations. Instead, we selected the following most significant parameter, horizontal corrected antisaccade latency. The neuropsychological evaluation was available in all controls and bvFTD patients, but only in 10 Alzheimer's disease and five svPPA patients. Due to the small number, svPPA was not included in this sub-analysis.

As a measure of global cognition, MMSE showed mild to moderate correlations with prosaccade and corrected antisaccade latencies and the percentage of correct memory saccades, especially in Alzheimer's disease and controls (Table 5).

**Table 5:** Relationship between neuropsychological performance and oculomotor parameters.

**Test**	**Prosaccade horizontal latency (*****r)***			**Corrected antisaccade horizontal latency (*****r)***			**Horizontal pursuit error (*****r)***			**% Correct memory saccades (*****r)***		
	***Crude P-value (adjusted P-value)***			***Crude P-value (adjusted P-value)***			***Crude P-value (adjusted P-value)***			***Crude P-value (adjusted P-value)***		
	**Controls**	**Alzheimer's disease**	**BvFTD**	**Controls**	**Alzheimer's disease**	**BvFTD**	**Controls**	**Alzheimer's disease**	**BvFTD**	**Controls**	**Alzheimer's disease**	**BvFTD**
MMSE (0–30)	−0.64	−0.58	−0.13	−0.36	−0.55	−0.23	0.014	−0.0062	−0.037	0.29	0.049	0.17
	***0.00032 (0.013)***	***0.012** (0.11)*	*0.65 **(0.030)***	*0.058 **(0.0061)***	***0.049** (0.065)*	*0.42 (0.76)*	*0.94 (0.93)*	*0.98 (0.89)*	*0.88 (0.31)*	*0.14 **(0.028)***	*0.89 (0.89)*	*0.57 (0.50)*
FCSRT Total Free and Cued Recall (0–48)	−0.0079	0.12	−0.22	−0.32	0.29	0.016	−0.067	−0.15	−0.36	0.24	0.90	0.14
	*0.97 (0.43)*	*0.75 (0.48)*	*0.42 (0.32)*	*0.10 (0.21)*	*0.53 (0.96)*	*0.96 (0.47)*	*0.75 (0.88)*	*0.68 (0.062)*	*0.14 (0.36)*	*0.22 (0.41)*	***0.037 (0.016)***	*0.63 (0.78)*
FCSRT Delayed Free and Cued Recall (0–16)	−0.078	0.12	−0.081	−0.20	0.14	−0.03	−0.22	0.062	−0.40	0.19	0.87	0.18
	*0.70 (0.75)*	*0.73 (0.45)*	*0.77 (0.37)*	*0.31 (0.30)*	*0.76 (0.75)*	*0.91 (0.60)*	*0.27 (0.18)*	*0.87 (0.16)*	*0.10 (0.17)*	*0.34 (0.74)*	*0.053 **(0.00031)***	*0.55 (0.65)*
ROCFT Recall (0–36)	−0.20	−0.15	−0.27	−0.22	0.25	0.21	−0.25	−0.48	−0.32	−0.026	0.89	0.26
	*0.31 (0.69)*	*0.72 (0.81)*	*0.31 (0.26)*	*0.26 (0.11)*	*0.64 (0.58)*	*0.49 (0.30)*	*0.23 (0.33)*	*0.23 **(0.038)***	*0.22 (0.53)*	*0.90 (0.92)*	***0.041** (0.054)*	*0.38 (0.50)*
ROCFT Copy (0–36)	−0.058	−0.71	−0.62	−0.0053	−0.99	−0.28	−0.19	−0.35	−0.49	0.42	−0.41	0.041
	*0.77 (0.81)*	*0.050 **(0.00092)***	***0.010 (0.0063)***	*0.98 (0.085)*	***0.00031** (0.17)*	*0.35 (0.90)*	*0.35 (0.42)*	*0.40 (0.37)*	***0.045 (0.0043)***	***0.026** (0.053)*	*0.49 (0.63)*	*0.88 (0.84)*
Imitative praxis (0–8)	0.29	−0.72	−0.52	0.000	−0.40	−0.097	−0.26	−0.36	−0.22	0.19	0.71	−0.30
	*0.17 (0.30)*	***0.020 (0.0019)***	*0.073 **(0.038)***	*1.00 (0.99)*	*0.38 (0.43)*	*0.77 (0.85)*	*0.22 (0.16)*	*0.32 (0.42)*	*0.44 (0.43)*	*0.34 (0.80)*	*0.18 (0.48)*	*0.37 (0.77)*
VOSP NL (0–10)	−0.13	−0.084	0.014	−0.16	−0.76	0.21	−0.10	−0.16	−0.45	0.10	−0.20	0.16
	*0.52 (0.85)*	*0.84 (0.19)*	*0.96 (0.96)*	*0.42 (0.11)*	***0.046** (0.18)*	*0.47 (0.75)*	*0.61 (0.42)*	*0.71 (0.91)*	*0.059 (0.41)*	*0.61 (0.73)*	*0.75 (0.71)*	*0.60 (0.56)*
Trail making test A (seconds)	0.15	0.49	0.32	0.50	0.82	−0.033	0.11	0.16	0.45	−0.20	−0.30	0.33
	*0.46 (0.30)*	*0.15 (0.31)*	*0.23 **(0.012)***	***0.0074 (0.0062)***	***0.024** (0.10)*	*0.91 (0.90)*	*0.60 (0.10)*	*0.65 (0.55)*	*0.062 (0.21)*	*0.32 (0.99)*	*0.62 (0.17)*	*0.25 (0.50)*
Trail making test B (seconds)	0.55	–	0.30	0.64	–	0.20	0.25	–	0.30	0.00	–	0.11
	***0.0038** (0.21)*		*0.62 (0.32)*	***0.00037** (**0.000025**)*		*0.75 (0.37)*	*0.24 (0.18)*		*0.62 (0.52)*	*1.00 (0.99)*		*0.90 (0.97)*
Symbol digit test	−0.61	−0.054	−0.25	−0.60	−0.60	0.070	−0.27	−0.16	−0.63	0.052	0.80	0.19
	***0.0031 (0.015)***	*0.90 (0.68)*	*0.36 (0.29)*	***0.0040 (0.0016)***	*0.29 (0.22)*	*0.81 (0.24)*	*0.24 **(0.046)***	*0.73 (0.59)*	***0.0051 (0.023)***	*0.81 (0.25)*	*0.20 (0.34)*	*0.52 (0.72)*

*Bold values indicate P < 0.05. Trail Making Test B could not be calculated in Alzheimer's disease sample since it was carried out in only two of these patients. BvFTD, behavioral variant frontotemporal dementia; FCSRT, Free and Cued Selective Reminding Test; MMSE, Mini-Mental State Examination; ROCFT, Rey-Osterrieth Complex Figure Test; VOSP NL, Visual Object and Space Perception Battery, Number Location subtest. r, correlation coefficient; P, level of significance*.

Prosaccade latencies showed moderate to strong negative correlations with ROCFT copy and imitative praxis both in Alzheimer's disease and bvFTD patients and with Symbol digit test in controls, meaning that longer latencies were associated with worse cognitive performance. Regarding corrected antisaccade latencies, moderate correlations were found with executive function tests in controls, including TMT-A and B and Symbol digit test. Alzheimer's disease patients also showed strong correlations between corrected antisaccade latencies and TMT-A and ROCFT copy, but it lost significance in the multivariate analysis. Pursuit error was negatively correlated with the Symbol digit test in bvFTD and controls, and also with ROCFT recall and copy in Alzheimer's disease and bvFTD groups, respectively. Finally, correct memory saccades were the only oculomotor parameter associated with verbal memory, strongly correlated with Total Free and Delayed Recall and borderline significant to visual recall in Alzheimer's disease patients.

### Relationship Between Oculomotor Behavior and Regional Brain Metabolism

**Figure 5:**
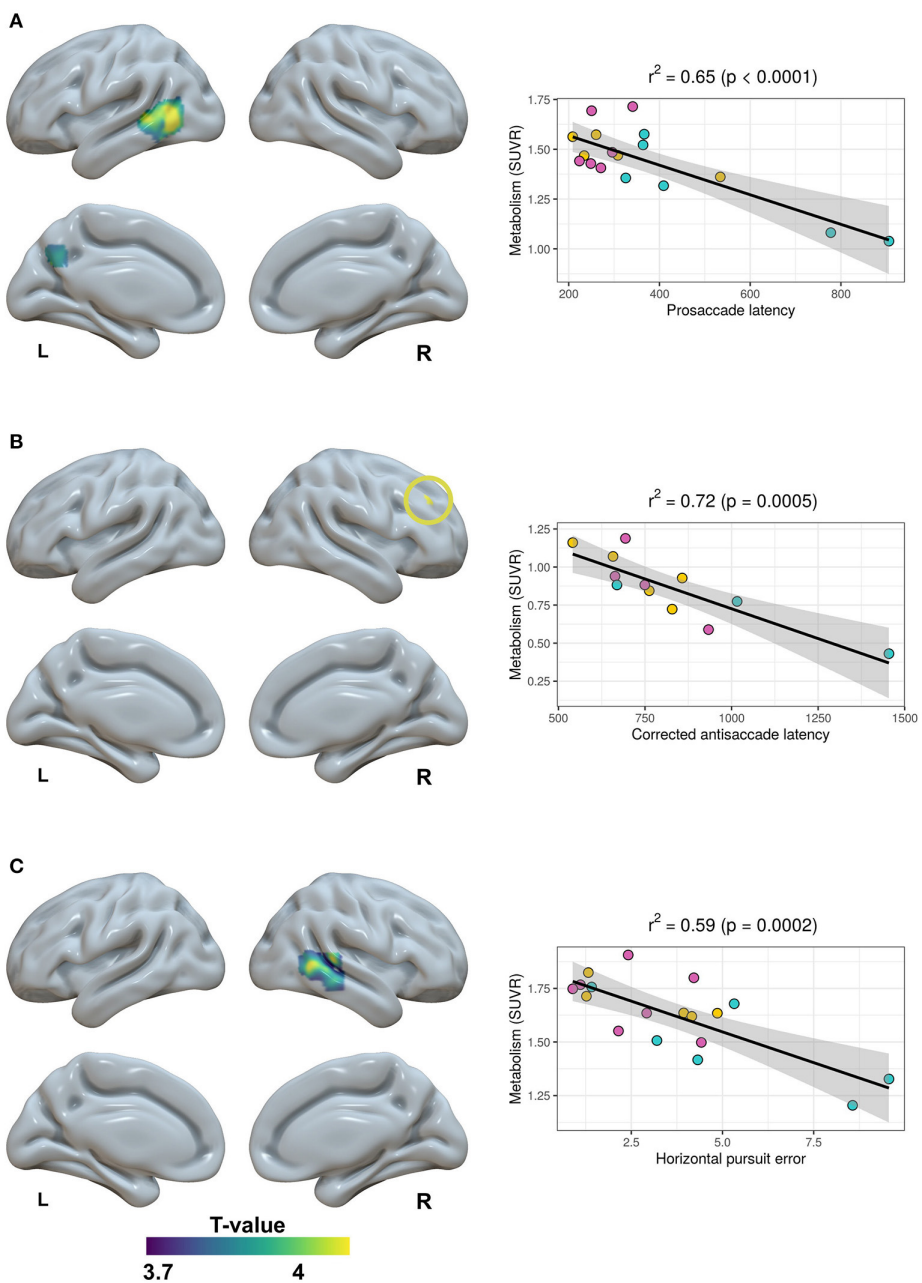
Relationship between brain metabolism and horizontal prosaccade latency **(A)**, horizontal corrected antisaccade latency **(B)** and horizontal pursuit error **(C)**. SUVR, Standard Uptake Value Rate.

18F-FDG PET was available for 18 patients (six Alzheimer's disease, seven bvFTD, and five svPPA). We investigated for negative associations with horizontal prosaccade latency, horizontal pursuit error and horizontal corrected antisaccade latency; and for positive associations with the percentage of correct memory saccades. Brain regions that showed significant associations at an uncorrected threshold of *P* < 0.001 (k>100 mm3) are depicted in Figure 5.

Prosaccade latency was negatively correlated with the metabolism in the left middle temporal gyrus and precuneus. This association was mainly driven by Alzheimer's disease cases (see scatterplot on Figure 5A). A greater corrected antisaccade latency was associated with decreased metabolism in the right middle frontal gyrus. Pursuit error was negatively correlated with the metabolism in the right posterior middle temporal gyrus. In both cases, the association was more uniformly distributed over the three disease groups. Finally, no significant associations were found with the percentage of correct memory saccades.

### Classification Algorithm

Each algorithm was trained using those oculomotor parameters that, after the normalization process, demonstrated significant differences between groups in the univariate analysis at a significance level of *P* < 0.001. Furthermore, parameters that were susceptible to having unavailable values, as time or accuracy-related parameters of correct antisaccades or memory saccades, were excluded. The oculomotor parameters that were finally included in each algorithm can be found in the Supplementary Material.

Based on the accuracy of each algorithm, we selected the best classifier with higher values in ROC curves, which were: Supported Vector Machine for the Alzheimer's disease vs. controls and bvFTD vs. controls pairs; and K-Nearest Neighbors for the Alzheimer's disease vs. bvFTD pair. The cross-validation under C++ environment offered the final results, which are summarized in Table 6. The classifiers demonstrated a mean AUC of 97.5% for the differentiation between Alzheimer's disease vs. controls, 96.7% for bvFTD vs. controls, and 92.5% for Alzheimer's disease vs. bvFTD.

**Table 6:** Performance of the machine learning classifiers.

**Pairs of groups**	**Best AUC**	**Worst AUC**	**Mean AUC**	**Best accuracy**	**Worst accuracy**	**Mean accuracy**	**Selected machine learning algorithm**
Alzheimer's disease vs. controls	0.9985	0.95	0.9753	0.973	0.8649	0.9478	Supported vector machine
bvFTD vs. controls	0.9912	0.9147	0.9674	0.9459	0.8919	0.9323	Supported vector machine
Alzheimer's disease vs. bvFTD	0.9722	0.7778	0.9246	0.9722	0.7778	0.9246	k-Nearest neighbors

*AUC, area under the curve; BvFTD, behavioral variant frontotemporal dementia*.

The classifiers for the differentiation between controls and each dementia group were subsequently applied in the independent samples of Alzheimer's disease and bvFTD to test their external validity. The independent sample of Alzheimer's disease patients was composed of 15 individuals without a biomarker-supported diagnosis who were in a mild dementia stage (Global Deterioration Scale = 4) as the discovery sample and showed comparable MMSE scores and number of sedatives, but were significantly older (mean difference 9.50 years, *P* = 0.00022) (Table 7). The AUC in this sample was 73.3%. For the replication of the bvFTD vs. controls algorithm, the independent sample included six patients with similar age, disease duration, MMSE and number of sedatives to that of the discovery sample. A biomarker-supported diagnosis was available in two cases. Here, the AUC was 83.3%.

**Table 7:** Demographics of Alzheimer's disease and bvFTD independent samples.

	**Alzheimer's disease independent sample**		**BvFTD independent sample**	
	**Characteristic**	**Mean difference from discovery sample (*P*-value)**	**Characteristic**	**Mean difference from discovery sample (*P*-value)**
*n*	15		6	
Age, years	77.67 (5.90)	9.50 ***(0.00022)***	71.40 (3.78)	2.57 *(0.53)*
Gender, % female	53.33	– *(0.49)*	40.00	– *(0.58)*
Disease duration, years	3.91 (1.38)	−1.04 *(0.10)*	2.75 (1.71)	−2.81 *(0.10)*
Intake of sedatives, mean number	0.86 (0.86)	−0.20 *(0.53)*	1.50 (0.71)	−0.56 *(0.50)*
MMSE (0–30)	19.27 (3.71)	2.54 *(0.12)*	25.00 (2.55)	1.50 *(0.28)*

*Mean values of each demographic characteristic with standard deviations in brackets () and mean differences in comparison with the discovery sample with level of significance (P-value), calculated with T-test. Bold values indicate P < 0.05. BvFTD, behavioral variant frontotemporal dementia; MMSE, Mini-Mental State Examination; svPPA, semantic variant of primary progressive aphasia. r, correlation coefficient; P, level of significance*.

## Discussion

**Figure 6:**
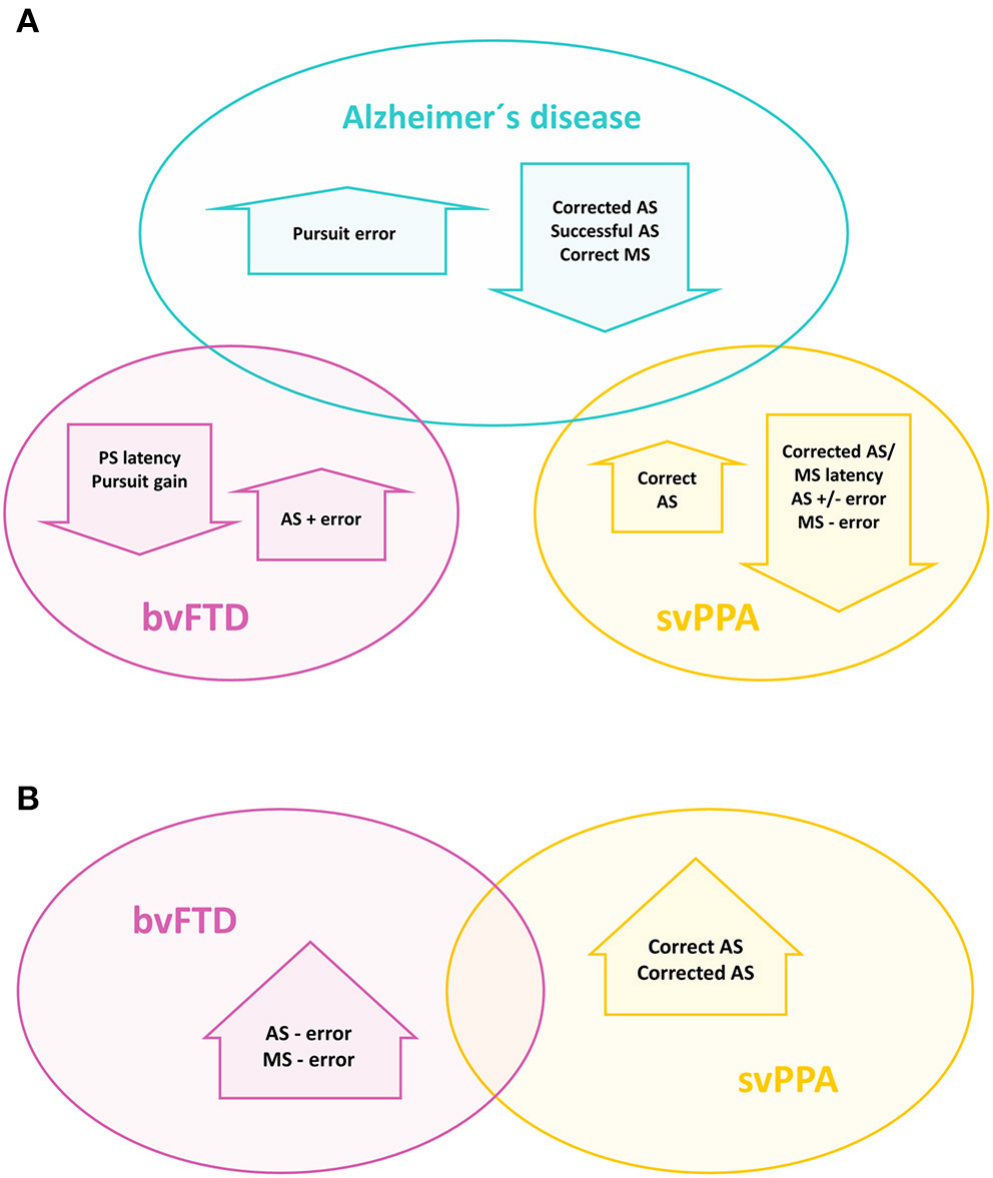
Useful oculomotor parameters for the differential diagnosis between Alzheimer's disease and the other two dementia groups **(A)**, and between bvFTD and svPPA **(B)**. AS, antisaccade; MS, memory saccade; PS, prosaccade.

In this work, we have compared the oculomotor behavior of Alzheimer's disease, bvFTD and svPPA patients, evaluated with a new video-oculography system and quantitatively described by a wide range of parameters, grouped through the axes of spatial accuracy, time and success. Their assessment across the four oculomotor tests offered a pattern that could differentiate each group from the others (Figure 6) and reflected the characteristic neuroanatomical distribution of pathology of each disease. Alzheimer's disease was the most impaired group in the three axes and, as expected, displayed specific impairments in some visually-guided saccade parameters that are theoretically closely linked to posterior brain regions, as pursuit error and horizontal prosaccade latency. Conversely, bvFTD patients showed deficits especially in the more challenging volitional saccades, which require a fine control exerted mainly from frontal lobe regions; while svPPA patients performed in general similarly to controls except from a lower success rate in the memory saccade test.

### Parameters Related to Spatial Accuracy: Pursuit Error Distinguishes Alzheimer's Disease

Alzheimer's disease patients showed the least accurate saccades, tending to obtain the highest values of spatial error not only in their volitional saccades but also in their prosaccades, which could suggest a more impaired visuospatial function. However, probably one of the most remarkable findings of this work is their performance on the smooth pursuit test, where these patients obtained significantly higher pursuit error values than the other three groups. Reported findings in Alzheimer's disease patients in this test are decreased velocity or gain (Molitor et al., 2015). The two studies that have investigated neuroimaging correlates in patients with dementia did not find any association with a specific cortical region (Boxer et al., 2006; Shakespeare et al., 2015), but these studies assessed other pursuit parameters than pursuit error. According to current literature, the middle temporal complex (also named V5), a region localized bilaterally in lateral occipitotemporal cortex (Coiner et al., 2019), constitutes the key cortical area for smooth pursuit since it is implicated in extracting motion information from the moving target and afterwards sending it to pursuit-specific regions of the frontal and supplementary eye fields (Petit and Haxby, 1999; Krauzlis, 2004; Thier and Ilg, 2005). Here, we found that pursuit error was associated with decreased metabolism in the right posterior middle temporal gyrus, in a region located closely to middle temporal complex. One possibility is that this absence of complete concordance could be related to the relatively low inaccuracy of the spatial normalization and resolution of the PET. In this way, increased values of pursuit error might be considered as a surrogated marker of posterior cortical damage and therefore be suggestive of Alzheimer's disease pathology in a given patient, as opposed to other conditions that predominantly harm frontal lobe structures.

### Parameters Related to Time: Different Meanings of Visually-Guided and Volitional Saccade Latencies

Alzheimer's disease patients also showed longer mean latencies than the other three groups across all paradigms, which replicates one of the most characteristic findings in the literature. Lesion studies have traditionally linked an increment in prosaccade latencies to parietal cortex damage, a region considered to play an important role in saccade triggering, supported by the existence of direct projections to the superior colliculus (Pierrot-Deseilligny et al., 1991; Gaymard et al., 2003). Similarly, in Alzheimer's disease samples, it has been related to increased parietal atrophy (Shakespeare et al., 2015). In our study, prosaccade latencies showed the strongest correlations to constructional and imitative praxis in the neuropsychological evaluation, which supports this hypothesis. The associations with brain metabolism involved the left middle temporal and precuneus. Precuneus is considered to be part of the oculomotor network (Coiner et al., 2019) and has previously demonstrated functional activation during different saccade responses, including prosaccades (Jamadar et al., 2013). However, in our study, these associations seem to be driven by some Alzheimer's disease cases. Since these regions are those primarily targeted in Alzheimer's disease, we cannot exclude that this result reflects between-group differences rather than the metabolic substrates of prosaccade latency.

In the case of bvFTD, some previous works also observed significantly longer saccade latencies in comparison with healthy controls (Meyniel et al., 2005; Burrell et al., 2012; Douglass et al., 2018), while others not (Boxer et al., 2006, 2012; Garbutt et al., 2008). In our work, bvFTD patients showed prosaccade latencies comparable to those of controls, but longer than them for corrected antisaccades and memory saccades. This underlines the differences between the processing of visually-guided and volitional saccades, where latencies not only represent the time required to perceive a peripheral target and to generate a saccade, but also to build an internal spatial representation of the demanded saccade without visual reference and to process the intended response, steps which finally increase the global amount of time (McDowell et al., 2008). According to this, latencies for corrected antisaccades and memory saccades were prominently longer than for prosaccades in all groups, and especially in Alzheimer's disease and bvFTD. In controls, corrected antisaccade latencies showed significant correlations with TMT-A, Symbol digit test and, with the greatest coefficient, with TMT-B, a test that assesses visuomotor speed and cognitive flexibility, which agrees with previous works which have found associations between executive function and antisaccade performance (Meyniel et al., 2005; Heuer et al., 2013; Douglass et al., 2018). Previous publications have reported associations between antisaccade parameters and diverse frontal regions in patients with dementia and normal elders (Boxer et al., 2006; Mirsky et al., 2011). Here, we also found a strong negative correlation between corrected antisaccade latency and metabolism in a region of the right middle frontal gyrus located in the DLPFC. The DLPFC is considered to play a decisional role in the antisaccade task through the inhibition of prosaccades, as well as supporting working memory in maintaining the task goal, which allows performing a successful antisaccade (Pierrot-Deseilligny et al., 2003). Right lateralization of DLPFC activation during antisaccade tasks has been reported before (Ford et al., 2005; Ettinger et al., 2008; Jamadar et al., 2013). Considering that corrected antisaccade latency measures the time required to make a correction after an erroneous antisaccade, the association found with right DLPFC might be more related to working memory activity and task monitoring, which has been specifically linked to right DLPFC (Ford et al., 2005; Kaufman et al., 2010).

Contrarily to the findings in Alzheimer's disease and bvFTD, svPPA latencies of corrected antisaccades and memory saccades were comparable to controls and significantly shorter than in Alzheimer's disease. SvPPA constitutes an entity in that initial pathology tends to affect selectively anterior temporal poles, which leads to a specific impairment of semantic memory with a relative sparing of other cognitive abilities during the first stages of the disease. Considering that the reported cortical substrate of oculomotor behavior relies on a core frontoparietal network, it is not surprising that the three published works that offer disaggregated data from svPPA subjects (Boxer et al., 2006, 2012; Garbutt et al., 2008) have found performances indistinguishable from healthy controls. Therefore, we find intriguing that, in our study, svPPA patients tended to show longer prosaccade latencies, in contrast to their preserved volitional saccade latencies. It may be noted that Alzheimer's disease and svPPA patients made a total number of early saccades significantly greater than controls (see Supplementary Table 1), but statistically significant differences in prosaccade latencies remained significant after accounting for it. Since the generation of prosaccades is supported by a widespread network, one possible explanation is that, in svPPA patients, the increase in prosaccade latencies is not due to parietal damage, as reported in Alzheimer's disease, but by a disruption in other nodes of the neural circuitry. Supporting this idea is the fact that, in controls, prosaccade latencies were not correlated to constructive and imitative praxis as in Alzheimer's disease, but with the Symbol digit test, and, in bvFTD, also to TMT-A, both tests which assess executive function. Furthermore, in the work which includes the largest sample of svPPA with 19 subjects (Garbutt et al., 2008), prosaccade latencies showed significant correlations not only to parietal and occipital but also to right temporal volumes. Consequently, we may hypothesize that the same impairment in an oculomotor parameter could be due to different anatomic damage depending on the underlying condition. This also suggests that oculomotor parameters which rely on more widespread networks could be less useful for the differential diagnosis between pathologies, while parameters more tightly controlled by a particular brain region, as could be the case of pursuit error, might constitute better markers of a specific pathology.

### Parameters Related to Success: A Pattern of Impairment for Each Disease

The lowest success rates were found in the Alzheimer's disease group, with significantly lower percentages of successful antisaccades and correct memory saccades than the other three groups. According to the literature, the bvFTD group also made fewer correct antisaccades (Meyniel et al., 2005) and memory saccades (Douglass et al., 2018) than controls. Some previous works have described that bvFTD patients were able to correct as many errors as controls and more than Alzheimer's disease patients (Boxer et al., 2006; Garbutt et al., 2008), while others did not (Boxer et al., 2012; Douglass et al., 2018). Here, bvFTD patients exhibited an intermediate ability to correct antisaccade errors, significantly greater than the Alzheimer's disease group but not comparable to controls. In conclusion, bvFTD patients displayed a normal performance in the prosaccade test, but showed deficits, including longer latencies, greater values of spatial error and lower success rates, only in the more cognitively demanding volitional saccades, which implicate a supportive role from frontal regions as the DLPFC or anterior cingulate cortex (McDowell et al., 2008; Jamadar et al., 2013).

The performance of svPPA patients was comparable to controls in the antisaccade test, but lower in the memory saccade test. To our best knowledge, an evaluation of memory saccades in svPPA has not been described before, so we consider that this is a novel finding. This way, svPPA could be differentiated from controls by the percentage of correct memory saccades; and from bvFTD and Alzheimer's disease patients by the percentage of correct and corrected antisaccades. Given that the instructions for the memory saccade test are more complicated than for the visually-guided tests, it is important to consider that the comprehension impairment that characterizes svPPA may be a limiting factor for the successful performance of this test. However, we think that, in our sample, this is less probable, since all svPPA patients were able to perform some correct memory saccades, with the lowest percentage of success of 20% in two patients. Moreover, this sample achieved high rates of success in the antisaccade test, which may be equally difficult to understand.

### Classification Algorithms

The last objective of this work was to test whether a classification algorithm based on machine learning techniques could be useful for the differential diagnosis of these diseases. Machine learning is a subfield of the artificial intelligence disciple that leverages numerical techniques to automatically “learn” programs for performing these tasks by processing a huge quantity of data. The use of machine learning algorithms applied to different medical data has increased in the last decade and, in particular, those applications related with medical images (De Fauw et al., 2018; Rajpurkar et al., 2018; Elaziz et al., 2020; Stemmer et al., 2020). However, machine learning techniques arise as a powerful tool to analyses data coming from different sources (Corey et al., 2018; Fontanella et al., 2018), in order to assist clinicians in the diagnosis stage (Kim et al., 2017; Viscaino et al., 2020). Moreover, a previous study described a machine learning model based on smooth pursuit data that discriminated age-matched controls from young-onset Alzheimer's disease patients with ~95% accuracy (Pavisic et al., 2017). In our work, we incorporated the most significant parameters from the four oculomotor tasks to train algorithms that were able to distinguish Alzheimer's disease, bvFTD, and controls. These results were replicated in independent samples, and, in the case of bvFTD patients, also from a different memory clinic. Although the classification accuracy of the algorithm for differentiating Alzheimer's disease from controls was lower in the replication sample, it must be taken into consideration that this group lacked biomarkers. Thus, it was potentially a more heterogeneous sample, posing a greater challenge to the classification algorithm, but that at the same time it was a more representative sample of everyday practice and therefore a more rigorous test of its external validity. Additionally, the discovery sample was composed of younger patients that tended to be more cognitively impaired, with non-significantly lower MMSE scores. Despite this, the algorithm was able to identify 11 out of 15 patients of the replication sample. All in all, our results should be considered only as an encouraging first step toward the application of oculomotor evaluations in medical practice, where machine learning approaches offer the possibility of incorporating information from a high number of parameters.

### Limitations

A potential limitation of this study is the small sample sizes, especially in the case of svPPA patients, where results must be taken with caution. In spite of this, most findings are concordant with previous publications, and we think that these results are reinforced by the fact that exclusively patients with Alzheimer's disease core biomarker-supported diagnoses were included, which, to our best knowledge, is the first time both for the case of Alzheimer's disease and FTD literature. The only previous work in FTD patients with available Alzheimer's disease core biomarkers reported the results of CSF biomarkers in only six out of 14 patients, and oculomotor information was not disaggregated from the whole sample that included bvFTD, svPPA, non-fluent progressive aphasia, and motor neuron disease-FTD (Coppe et al., 2012). Also, to maximize homogeneity in our discovery sample, all included patients were in the same functional stage and showed similar disease duration, a relevant factor considering that it has been described that some oculomotor parameters become increasingly impaired with disease progression (Molitor et al., 2015; Kahana Levy et al., 2018).

Additionally, an issue that might limit clinical translation is that some patients might not be able to fully understand the most cognitively demanding tasks, the antisaccade and memory saccade tests. However, visually-guided saccade tests, which could be used in moderately-demented patients, were also useful in differentiating Alzheimer's disease from the other groups.

Finally, we reported neuroimaging results at an uncorrected threshold, since the 18F-FDG PET evaluation was available only in 18 patients and our statistical power was low. Additionally, we cannot exclude that the associations found between prosaccade latency and brain metabolism are driven by some Alzheimer's disease cases, since increased latencies and hypometabolism in middle temporal gyrus and precuneus can be a shared finding in this disease. Conversely, the results regarding corrected antisaccade latency and pursuit error are concordant with the current literature since they involve well-known related core regions of the oculomotor system, such as the DLPFC and the posterior middle temporal gyrus. In any case, we recommend extreme caution in the interpretation of the brain metabolism results and we suggest considering them as supportive findings of the oculomotor outcomes rather than firm conclusions about the neuroanatomic correlates of oculomotor parameters.

### Conclusion and Future Works

In this work, we have described differential patterns in oculomotor behavior in typical Alzheimer's disease, bvFTD and svPPA and introduced a machine learning methodology that could be applied in clinical practice to patients who often pose a diagnostic challenge. Some of the reported findings, especially those related to success parameters, are also accessible for routine clinical examination. Further works should address whether some oculomotor features are early enough to uncover the presence of neurodegenerative changes in prodromal stages, and specific enough of the characteristic spreading of cortical and network neurodegeneration of each disease to distinguish them even when the clinical picture is less defined, as atypical presentations of Alzheimer's disease. Furthermore, it would be of great interest to analyse whether reported correlations between oculomotor parameters and neuroimaging in healthy controls and lesion studies are the same in neurodegenerative diseases, due to the deep complexity of the neuroanatomic substrates of oculomotor behavior.

## Data Availability Statement

The original contributions presented in the study are included in the article/Supplementary Material, further inquiries can be directed to the corresponding author/s.

## Ethics Statement

The studies involving human participants were reviewed and approved by Comité de Ética de Investigación Clínica de Cantabria (CEIC), Marqués de Valdecilla Health Research Institute. The patients/participants provided their written informed consent to participate in this study.

## Author Contributions

CL: study design, oculomotor evaluations, data analysis, and drafting of the manuscript. SL-G, MK, and IA-B: oculomotor evaluations and collection of clinical data. AB: neuroimaging data analysis. AC-C: oculomotor data processing and artificial intelligence procedures. AP and MG-M: neuropsychological assessments. AF-R and MB-G: collection of biological samples and coordination of subject recruiting. JJ-B and IB: neuroimaging evaluations. JI-V: CSF analytical procedures. JP: neuroimaging processing. CG-C: study concept and design and artificial intelligence procedures. PS-J: study concept and design and statistical advisement. ER-R, AL-B, JF, II-G, and all authors: critical revision of the manuscript.

## Conflict of Interest

The authors declare that the research was conducted in the absence of any commercial or financial relationships that could be construed as a potential conflict of interest.

## Abbreviations

AUC: area under the curve
bvFTD: behavioral variant frontotemporal dementia
DLPFC: dorsolateral prefrontal cortex
18F-FDG: 2-[18F] Fluoro-2-Deoxy-D-Glucose
MMSE: Mini-Mental State Examination
PiB: Pittsburgh Compound-B
ROC: receiver operating characteristic
ROCFT: Rey-Osterrieth Complex Figure Test
TMT: Trail Making Test
svPPA: semantic variant of primary progressive aphasia.
