# Supplementary material for: Distinctive Oculomotor Behaviors in Alzheimer's Disease and Frontotemporal Dementia
Source: Front Aging Neurosci. 2021 Feb 4;12:603790. doi: 10.3389/fnagi.2020.603790 (PMC7891179; doi:10.3389/fnagi.2020.603790)
Supplement: Supplementary file 1 [file Table_1.DOCX]

Supplementary Material

**Index**

1. **Oculomotor evaluation**
   1. **Eye-tracking technology**
   2. **Oculomotor assessment**
   3. **Definitions of oculomotor parameters**
2. **Machine learning classification algorithms**
3. **Extended results**
   1. **Supplementary table 1: Prosaccade test**
   2. **Supplementary table 2: Antisaccade test**
   3. **Supplementary table 3: Memory saccade test**
4. **Oculomotor evaluation**
   1. **Eye-tracking technology**

The oculomotor evaluation was carried out with OSCANN, an eye-tracking sensor based on video electro-oculography. Eye movements are recorded by a high-speed infrared camera that can store images at a rate of 100 frames per second. The device includes a forehead and chin rests to ensure head stability during the tasks, when participants remain seated in front of a display at a viewing distance of 60 cm. Processing of the registered data was made offline with the software provided and a posterior analysis was carried out in a semi-automated manner by personnel from Aura, who was blinded to clinical diagnoses.

- 1. **Oculomotor assessment**

Assessments were conducted in a quiet dark room. Each test was preceded by a calibration trial, in which one central target and eight eccentric targets appeared subsequently on the display during 1500 ms (milliseconds) each. After calibration, tests began with a central fixation target which was followed by 12 trials in the horizontal plane and 8 trials in the vertical plane. For the prosaccade and antisaccade tests, eccentric targets remained fixed for 3000 ms and then were immediately replaced by a central target for other 3000 ms. In the memory saccade test, the duration of both the stimuli and the blank period for the performance of the memory saccade was also 3000 ms.

The sequence of tests was: prosaccade test; antisaccade test; sinusoidal smooth pursuit test; memory saccade test.

The overall duration of the evaluation was 20-25 minutes.

- 1. **Definitions of oculomotor parameters**
     1. **Parameters related to spatial accuracy**
- Spatial error: Difference between the position of the target and the final position of the eye. Positive error values are the result of eye positions more eccentric than the target, and negative values of eye positions more central than the target.
- Pursuit error: Difference between the target position and the gaze position during the SSPT.
  - 1. **Parameters related to time**
- Latency: Time between the appearance of an eccentric target and the onset of a first ocular movement which cannot be considered an early saccade (see below at *1.3.3.Parameters related to success and early saccades*).
- Corrected antisaccade duration: Time between the onset of the eye fixation in an erroneous antisaccade and the onset of the eye fixation in a subsequent corrected antisaccade.
- Peak eye velocity: The highest value of eye velocity during a saccadic movement.
- Pursuit gain: Rate between the ocular velocity and the target velocity during the SSPT.
  - 1. **Parameters related to success and early saccades**
- Correct antisaccade: Saccadic movement made in the opposite direction from the appearing target which starts from the central target of the screen.
- Percentage of correct antisaccades: Percentage of correct antisaccades from the total number of trials.
- Erroneous antisaccade: Saccadic movement made towards the target in an antisaccade test.
- Percentage of erroneous antisaccades: Percentage of erroneous antisaccades from the total number of trials.
- Corrected antisaccade: Saccadic movement made in the opposite direction from the appearing target after an erroneous antisaccade and which at least exceeds the centre of the screen.
- Percentage of corrected antisaccades: Percentage of corrected antisaccades from the total number of erroneous antisaccades.
- Successful antisaccades: Sum of correct and corrected antisaccades.
- Percentage of successful antisaccades: Percentage of successful antisaccades from the total number of trials.
- Uncorrected erroneous antisaccade: Erroneous antisaccade which is not followed by a corrected antisaccade.
- Percentage of uncorrected erroneous antisaccades: Percentage of uncorrected erroneous antisaccades from the total number of trials.
- Second order antisaccades: Saccadic movement made towards the target after a correct antisaccade and which at least exceeds the centre of the screen.
- Percentage of second-order antisaccades: Percentage of second-order antisaccades from the total number of trials.
- Correct memory saccade: Saccadic movement made towards the location in which the target had appeared previously and after the screen had become blank, in a memory saccade test.
- Percentage of correct memory saccades: Percentage of correct memory saccades from the total number of trials.
- Early saccade: Saccadic movement triggered in an interval of 80 ms after the appearance of an eccentric target. According to current literature, this period constitutes the minimum human time frame to generate a prosaccade in reaction to changes in the visual field, so therefore any saccade generated during it cannot be related to the appearing target.
- Percentage of early saccades: Percentage of early saccades from the total number of trials in a prosaccade, antisaccade or memory saccade test.

1. **Machine learning classification algorithms**

The oculomotor parameters which were finally included in the machine learning classifiers are listed below.

- 1. **Alzheimer’s disease versus controls algorithm**
- Latency of horizontal prosaccades (ms)
- Horizontal pursuit error (º)
- Number and percentage of correct horizontal antisaccades
- Number and percentage of uncorrected erroneous horizontal antisaccades
- Number and percentage of uncorrected erroneous vertical antisaccades
- Number and percentage of corrected horizontal antisaccades
- Percentage of corrected horizontal antisaccades over total trials
- Percentage of corrected vertical antisaccades
- Number and percentage of successful horizontal antisaccades
- Number and percentage of successful vertical antisaccades
- Number and percentage of early saccades in the horizontal antisaccade test
- Number and percentage of correct horizontal memory saccades
- Number and percentage of correct vertical memory saccades
  1. **BvFTD versus controls algorithm**
- Number and percentage of correct horizontal antisaccades
- Number and percentage of correct horizontal memory saccades
- Number and percentage of correct vertical memory saccades
  1. **Alzheimer’s disease versus bvFTD algorithm**
- Horizontal pursuit error (º)
- Horizontal return antisaccade peak velocity (º/ms)

1. **Extended results**

This section shows the results of additional oculomotor parameters obtained in the prosaccade test, the antisaccade test and the memory saccade test, not included in the tables displayed in the main text.

| **Supplementary table 1. Prosaccade test** | | | | | | | |
| --- | --- | --- | --- | --- | --- | --- | --- |
| **Oculomotor Parameter** | **Controls** | **Alzheimer´s disease vs controls**  *Crude P-value*  *(adjusted P-value)* | **BvFTD vs controls**  *Crude P-value*  *(adjusted P-value)* | **SvPPA vs controls**  *Crude P-value (adjusted P-value)* | **Alzheimer´s disease vs bvFTD**  *Crude P-value (adjusted P-value)* | **Alzheimer´s disease vs svPPA**  *Crude P-value (adjusted P-value)* | **BvFTD vs svPPA**  *Crude P-value (adjusted P-value)* |
| **Prosaccade peak velocity (º/ms)** |  |  |  |  |  |  |  |
| Horizontal mean value (SD) | 323.34 (62.43) | 360.79 (108.23)  *0.44 (0.13)* | 359.1 (78.28)  *0.51 (0.30)* | 392.43 (84.61)  *0.20* ***(0.046)*** | *1.00 (0.80)* | *0.82 (0.36)* | *0.81 (0.29)* |
| Vertical mean value (SD) | 247.16 (53.53) | 268.92 (52.66)  *0.57 (0.10)* | 263.14 (61.26)  *0.78 (0.30)* | 287.81 (39.59)  *0.30* ***(0.024)*** | *0.99 (0.69)* | *0.87 (0.29)* | *0.74 (0.18)* |
| **Return saccade peak velocity (º/ms)** |  |  |  |  |  |  |  |
| Horizontal mean value (SD) | 325.32 (73.18) | 382.99 (92.33)  *0.12* ***(0.038)*** | 377.84 (79.09)  *0.21 (0.13)* | 417.15 (119.91)  *0.061* ***(0.022)*** | *1.00 (0.78)* | *0.80 (0.41)* | *0.74 (0.32)* |
| Vertical mean value (SD) | 249.52 (57.83) | 263.94 (47.17)  *0.80* ***(0.016)*** | 243.40 (41.85)  *0.98 (0.10)* | 277.91 (54.96)  *0.56* ***(0.025)*** | *0.65 (0.62)* | *0.93 (0.62)* | *0.45 (0.38)* |
| **Early saccades (%)** |  |  |  |  |  |  |  |
| Total mean value (SD) | 1.55 (2.90) | 7.41 (9.31)  ***0.047 (0.0085)*** | 5.00 (9.35)  *0.42 (0.092)* | 10.00 (8.17)  ***0.038 (0.0067)*** | *0.76 (0.54)* | *0.86 (0.40)* | *0.43 (0.20)* |
| Horizontal mean value (SD) | 0.89 (2.63) | 9.26 (11.75)  ***0.0030 (0.00049)*** | 4.17 (6.09)  *0.52 (0.076)* | 11.91 (10.60)  ***0.0058 (0.0012)*** | *0.22 (0.20)* | *0.86 (0.44)* | *0.12 (0.083)* |
| Vertical mean value (SD) | 2.32 (6.04) | 2.94 (5.46)  *0.99 (0.76)* | 6.62 (15.38)  *0.47 (0.13)* | 7.14 (9.84)  *0.63 (0.19)* | *0.68 (0.25)* | *0.76 (0.30)* | *1.00 (0.94)* |

**Bold values indicate *P* < 0.05.**

**BvFTD** **= behavioural variant frontotemporal dementia;** **SD = Standard deviations; svPPA = semantic variant of primary progressive aphasia; º/ms = degrees per millisecond.**

| **Supplementary table 2. Antisaccade test** | | | | | | | |
| --- | --- | --- | --- | --- | --- | --- | --- |
| **Oculomotor Parameter** | **Controls** | **Alzheimer´s disease vs controls**  *Crude P-value*  *(adjusted P-value)* | **BvFTD vs controls**  *Crude P-value*  *(adjusted P-value)* | **SvPPA vs controls**  *Crude P-value (adjusted P-value)* | **Alzheimer´s disease vs bvFTD**  *Crude P-value (adjusted P-value)* | **Alzheimer´s disease vs svPPA**  *Crude P-value (adjusted P-value)* | **BvFTD vs svPPA**  *Crude P-value (adjusted P-value)* |
| **Correct antisaccades (%)** |  |  |  |  |  |  |  |
| Total mean value (SD) | 26.38 (18.75) | 4.00 (5.41)  ***0.00031 (0.00037)*** | 7.50 (10.80)  ***0.0022 (0.018)*** | 25.00 (29.33)  *1.00 (0.85)* | *0.93 (0.85)* | ***0.045 (0.016)*** | *0.12* ***(0.038)*** |
| Horizontal mean value (SD) | 27.30 (19.66) | 2.78 (5.14)  ***0.000075 (0.00016)*** | 5.21 (7.98)  ***0.00028 (0.013)*** | 22.22 (29.19)  *0.90 (0.59)* | *0.98 (0.82)* | *0.074* ***(0.025)*** | *0.14 (0.058)* |
| Vertical mean value (SD) | 25.00 (21.91) | 6.25 (10.69)  ***0.024 (0.0091)*** | 10.94 (16.38)  *0.11 (0.074)* | 29.17 (30.28)  0.97 *(0.78)* | *0.91 (0.93)* | *0.090* ***(0.033)*** | *0.22 (0.053)* |
| **Corrected antisaccades (%)** |  |  |  |  |  |  |  |
| Total mean value (SD) | 96.06 (11.11) | 46.08 (34.94)  ***1.61*10^-6^ (1.64*10^-6^)*** | 76.15 (33.96)  *0.057* ***(0.026)*** | 100.00 (0.00)  *0.98 (0.88)* | ***0.0066 (0.0496)*** | ***0.00016 (0.000058)*** | *0.19* ***(0.026****)* |
| Horizontal mean value (SD) | 98.42 (6.53) | 42.63 (37.48)  ***3.09 *10^-07^ (2.28*10^-07^)*** | 80.05 (37.11)  *0.11* ***(0.033)*** | 100.00 (0.00)  *1.00* ***(0.90)*** | ***0.00089 (0.015)*** | ***0.00013 (0.000042)*** | *0.38 (0.057)* |
| Vertical mean value (SD) | 94.68 (19.80) | 55.95 (41.03)  ***0.0014 (0.0013)*** | 74.48 (40.10)  *0.16 (0.19)* | 100.00 (0.00)  0.98 *(0.68)* | *0.36 (0.26)* | ***0.023 (0.0068)*** | *0.31 (0.11)* |
| **Successful antisaccades (%)** |  |  |  |  |  |  |  |
| Total mean value (SD) | 92.24 (14.92) | 36.44 (30.77)  ***1.36*10^-7^ (8.75*10^-8^)*** | 70.00 (34.88)  ***0.030 (0.0072)*** | 83.33 (16.63)  *0.86 (0.26)* | ***0.0024 (0.047)*** | ***0.0015 (0.00065)*** | *0.69 (0.12)* |
| Horizontal mean value (SD) | 93.10 (15.28) | 32.78 (31.88)  ***1.68*10^-07^ (8.02*10^-9^)*** | 70.31 (38.00)  ***0.046 (0.00026)*** | 77.78 (28.22)  *0.60 (0.085)* | ***0.0017 (0.020)*** | ***0.0063 (0.0025)*** | *0.94 (0.23)* |
| Vertical mean value (SD) | 90.95 (16.67) | 43.75 (37.90)  ***0.000020 (0.000013)*** | 69.53 (38.45)  *0.083* ***(0.040)*** | 91.67 (10.21)  *1.00 (0.88)* | *0.73 (0.18)* | ***0.0054 (0.0019)*** | *0.37 (0.069)* |
| **Uncorrected erroneous antisaccades (%)** |  |  |  |  |  |  |  |
| Total mean value (SD) | 1.21 (2.88) | 42.00 (32.23)  ***9.71*10^-07^ (9.88*10^-07^)*** | 17.19 (25.10)  *0.056* ***(0.028)*** | 0.00 (0.00)  *1.00 (0.97)* | ***0.0048 (0.037)*** | ***0.00025 (0.000078)*** | *0.28* ***(0.041)*** |
| Horizontal mean value (SD) | 0.862 (3.41) | 45.000 (34.04)  ***5.04*10^-07^ (4.54*10^-07^)*** | 13.021 (26.34)  *0.250* ***(0.080)*** | 0.00 (0.00)  *1.00 (0.90)* | ***0.00039 (0.0068)*** | ***0.00020 (0.000066)*** | *0.56* ***(0.12)*** |
| Vertical mean value (SD) | 1.724 (5.51) | 36.607 (38.44)  ***0.00062 (0.00044)*** | 23.438 (37.33)  ***0.044 (0.044)*** | 0.00 (0.00)  *1.00 (0.94)* | *0.51 (0.54)* | ***0.026 (0.0083 )*** | *0.24* ***(0.048 )*** |
| **Second order antisaccades (%)** |  |  |  |  |  |  |  |
| Total mean value (SD) | 1.21 (2.56) | 7.22 (9.21)  ***0.029 (0.0024)*** | 3.75 (6.95)  *0.61 (0.18)* | 10.83 (11.14)  ***0.010 (0.0015)*** | *0.47 (0.36)* | *0.67 (0.30)* | *0.13 (0.085)* |
| Horizontal mean value (SD) | 1.15 (2.92) | 5.56 (7.50)  *0.24 (0.087)* | 3.65 (10.08)  *0.70 (0.52)* | 13.89 (12.55)  ***0.0015 (0.00069)*** | *0.89 (0.41)* | *0.098* ***(0.030)*** | ***0.025 (0.0055)*** |
| Vertical mean value (SD) | 1.29 (3.87) | 9.82 (14.02)  ***0.015 (0.0015)*** | 3.91 (7.53)  *0.76 (0.79)* | 6.25 (10.46)  *0.56 (0.20)* | *0.24* ***(0.015)*** | *0.82 (0.31)* | *0.94 (0.32)* |
| **Early saccades (%)** |  |  |  |  |  |  |  |
| Total mean value (SD) | 4.14 (9.83) | 11.67 (12.63)  *0.21* ***(0.039)*** | 10.63 (14.25)  *0.32 (0.055)* | 10.00 (14.49)  *0.70 (0.17)* | *1.00 (0.59)* | *1.00 (0.90)* | *1.00 (0.57)* |
| Horizontal mean value (SD) | 2.87 (7.14) | 11.67 (10.82)  *0.17* ***(0.044)*** | 13.54 (18.73)  *0.055* ***(0.0054)*** | 13.89 (22.15)  *0.26* ***(0.050)*** | *0.98 (0.35)* | *0.99 (0.60)* | *1.00 (0.81)* |
| Vertical mean value (SD) | 6.04 (14.80) | 12.50 (20.22)  *0.57 (0.19)* | 6.25 (13.69)  *1.00 (0.94)* | 4.17 (6.46)  *0.99 (0.84)* | *0.69 (0.32)* | *0.69 (0.29)* | *0.99 (0.80)* |
| **Antisaccade latency (ms)** |  |  |  |  |  |  |  |
| Horizontal mean value (SD) | 526.40 (396.29) | 897.99 (519.99)  *0.33 (0.10)* | 710.66 (398.26)  *0.74 (0.23)* | 520.54 (84.43)  *1.00 (0.96)* | *0.89 (0.64)* | *0.61 (0.25)* | *0.91 (0.42)* |
| Vertical mean value (SD) | 467.92 (165.86) | 521.66 (103.91)  *0.96 (0.61)* | 542.82 (291.23)  *0.84 (0.21)* | 508.94 (254.40)  *0.98 (0.60)* | *1.00 (0.61)* | *1.00 (0.99)* | *0.99 (0.59)* |
| **Corrected antisaccade duration (ms)** |  |  |  |  |  |  |  |
| Horizontal mean value (SD) | 234.26 (166.77) | 1132.35 (829.20)  ***0.00018 (6.28*10^-05^)*** | 657.11 (940.67)  *0.14* ***(0.010)*** | 273.65 (112.37)  *1.00 (0.37)* | *0.18 (0.36)* | ***0.025 (0.0085)*** | *0.56 (0.092)* |
| Vertical mean value (SD) | 198.25 (68.91) | 925.53 (1060.10)  ***0.0016 (0.00018)*** | 527.10 (539.88)  *0.29* ***(0.032)*** | 209.76 (98.98)  *1.00 (0.53)* | *0.26 (0.47)* | *0.056* ***(0.023)*** | *0.65 (0.13)* |
| **Return antisaccade latency (ms)** |  |  |  |  |  |  |  |
| Horizontal mean value (SD) | 393.44 (94.79) | 528.45 (284.46)  *0.105* ***(0.020)*** | 525.62 (209.03)  *0.105* ***(0.014)*** | 407.19 (88.31)  *1.00 (0.69)* | *1.00 (0.73)* | *0.52 (0.24)* | *0.54 (0.15)* |
| Vertical mean value (SD) | 415.29 (176.67) | 408.76 (169.30)  *1.00 (0.90)* | 448.56 (224.15)  *0.94 (0.44)* | 498.98 (199.36)  *0.76 (0.31)* | *0.94 (0.43)* | *0.77 (0.31)* | *0.95 (0.71)* |
| **Return antisaccade peak velocity (º/ms)** |  |  |  |  |  |  |  |
| Horizontal mean value (SD) | 303.03 (88.08) | 278.31 (67.63)  0.82 *(0.55)* | 395.55 (93.80)  ***0.0079 (0.0031)*** | 394.64 (127.33)  *0.11* ***(0.014)*** | ***0.0029 (0.0017)*** | ***0.043 (0.0076)*** | *1.00 (0.92)* |
| Vertical mean value (SD) | 217.61 (74.99) | 292.99 (84.70)  ***0.017 (0.0055)*** | 241.31 (65.66)  *0.75 (0.77)* | 197.35 (85.00)  *0.93 (0.41)* | *0.26* ***(0.040)*** | *0.058* ***(0.0090)*** | *0.62 (0.32)* |

**Bold values indicate *P* < 0.05.**

**BvFTD** **= behavioural variant frontotemporal dementia;** **ms = milliseconds; SD = Standard deviations; svPPA = semantic variant of primary progressive aphasia; º/ms = degrees per millisecond.**

| **Supplementary table 3. Memory saccade test** | | | | | | | |
| --- | --- | --- | --- | --- | --- | --- | --- |
| **Oculomotor Parameter** | **Controls** | **Alzheimer´s disease vs controls**  *Crude P-value*  *(adjusted P-value)* | **BvFTD vs controls**  *Crude P-value*  *(adjusted P-value)* | **SvPPA vs controls**  *Crude P-value (adjusted P-value)* | **Alzheimer´s disease vs bvFTD**  *Crude P-value (adjusted P-value)* | **Alzheimer´s disease vs svPPA**  *Crude P-value (adjusted P-value)* | **BvFTD vs svPPA**  *Crude P-value (adjusted P-value)* |
| **Correct memory saccades (%)** |  |  |  |  |  |  |  |
| Total mean value (SD) | 94.14 (9.74) | 26.00 (19.41)  ***2.71*10^-11^ (1.22*10^-09^)*** | 40.36 (25.15)  ***1.01*10^-09^ (0.00064)*** | 64.17 (36.94)  ***0.0065 (0.025)*** | *0.30* ***(0.014)*** | ***0.0022 (0.00019)*** | *0.072 (0.14)* |
| Horizontal mean value (SD) | 92.82 (14.56) | 33.33 (25.76)  ***8.19 *10^-08^ (3.23*10^-06^)*** | 42.86 (26.72)  ***3.55*10^-07^ (0.039)*** | 63.89 (35.22)  ***0.027*** *(0.12)* | *0.73* ***(0.018)*** | ***0.050 (0.0036)*** | *0.23 (0.51)* |
| Vertical mean value (SD) | 95.83 (8.49) | 15.00 (18.45)  ***6.05*10^-12^ (1.39*10^-11^)*** | 36.61 (27.93)  ***4.66*10^-10^ (3.98*10^-05^)*** | 57.50 (41.07)  ***0.0018 (0.0023)*** | *0.064* ***(0.049)*** | ***0.0022 (0.000049)*** | *0.22 (0.067)* |
| **Early saccades (%)** |  |  |  |  |  |  |  |
| Total mean value (SD) | 8.56 (16.36) | 4.82 (9.91)  *0.92 (0.39)* | 14.41 (17.45)  *0.65 (0.91)* | 9.17 (10.21)  *1.00 (0.75)* | *0.47 (0.58)* | *0.95 (0.71)* | *0.90 (0.86)* |
| Horizontal mean value (SD) | 8.91 (20.89) | 5.21 (1.41)  *0.96 (0.52)* | 15.48 (21.15)  *0.72 (0.90)* | 6.94 (8.19)  *1.00 (0.55)* | *0.62 (0.71)* | *1.00 (0.97)* | *0.80 (0.67)* |
| Vertical mean value (SD) | 8.33 (14.29) | 5.36 (9.84)  *0.96 (0.55)* | 13.54 (14.56)  *0.71 (0.88)* | 15.00 (16.30)  *0.77 (0.62)* | *0.62 (0.76)* | *0.65 (0.36)* | *1.00 (0.53)* |
| **Return memory saccade latency (ms)** |  |  |  |  |  |  |  |
| Horizontal mean value (SD) | 396.39 (149.64) | 435.05 (308.89)  *0.97 (0.55)* | 368.89 (275.75)  *0.98 (0.78)* | 348.18 (147.64)  *0.96 (0.86)* | *0.92 (0.78)* | *0.90 (0.56)* | *1.00 (0.71)* |
| Vertical mean value (SD) | 381.18 (119.43) | 243.88 (127.12)  *0.22 (0.071)* | 337.63 (153.15)  *0.82 (0.41)* | 491.92 (157.74)  *0.40 (0.15)* | *0.63 (0.40)* | ***0.049 (0.016)*** | *0.22 (0.062)* |
| **Memory saccade peak velocity (º/ms)** |  |  |  |  |  |  |  |
| Horizontal mean value (SD) | 320.24 (73.54) | 247.29 (139.54)  *0.20 (0.24)* | 236.85 (76.98)  *0.050* *(0.38)* | 350.45 (110.29)  *0.90 (0.22)* | *0.99 (1.00)* | *0.20* ***(0.046)*** | *0.098 (0.062)* |
| Vertical mean value (SD) | 239.12 (67.02) | 165.57 (54.52)  *0.14 (0.051)* | 208.76 (101.31)  *0.67 (0.17)* | 191.96 (57.39)  *0.64 (0.29)* | *0.67 (0.61)* | *0.95 (0.61)* | *0.98 (0.96)* |
| **Return memory saccade peak velocity (º/ms)** |  |  |  |  |  |  |  |
| Horizontal mean value (SD) | 333.15 (95.80) | 280.69 (103.74)  *0.64 (0.52)* | 240.03 (102.55)  *0.050 (0.72)* | 364.08 (103.64)  *0.92 (0.21)* | *0.85 (0.89)* | *0.51 (0.11)* | *0.11 (0.12)* |
| Vertical mean value (SD) | 217.22 (74.71) | 387.61 (350.15)  *0.059* ***(0.015)*** | 196.82 (87.88)  *0.97 (0.72)* | 226.76 (80.77)  *1.00 (0.89)* | *0.060* ***(0.029)*** | *0.26 (0.078)* | *0.98 (0.69)* |

**Bold values indicate *P* < 0.05.**

**BvFTD** **= behavioural variant frontotemporal dementia;** **ms = milliseconds; SD = Standard deviations; svPPA = semantic variant of primary progressive aphasia; º/ms = degrees per millisecond.**
